# Understanding the Role of the SMN Complex Component GEMIN5 and Its Functional Relationship with Demethylase KDM6B in the Flunarizine-Mediated Neuroprotection of Motor Neuron Disease Spinal Muscular Atrophy

**DOI:** 10.3390/ijms251810039

**Published:** 2024-09-18

**Authors:** Badih Salman, Emeline Bon, Perrine Delers, Steve Cottin, Elena Pasho, Sorana Ciura, Delphine Sapaly, Suzie Lefebvre

**Affiliations:** 1 T3S, INSERM UMR1124, Faculté des Sciences Fondamentales et Biomédicales, Université Paris Cité, F-75006 Paris, France; badih.salman@inserm.fr (B.S.); emeline.bon@etu.u-paris.fr (E.B.); perrine.delers@u-paris.fr (P.D.); steve.cottin@gmail.com (S.C.); delphine.sapaly@u-paris.fr (D.S.); 2 INSERM UMR1163, Institut Imagine, Université Paris Cité, F-75015 Paris, France; elena.pasho@institutimagine.org (E.P.); sorana.ciura@institutimagine.org (S.C.)

**Keywords:** motor neuron disease, spinal muscular atrophy, RNA metabolism, SMN complex, GEMIN5, KDM6B, JMJD3, MNX1, HB9, FNIP1, flunarizine

## Abstract

Dysregulated RNA metabolism caused by SMN deficiency leads to motor neuron disease spinal muscular atrophy (SMA). Current therapies improve patient outcomes but achieve no definite cure, prompting renewed efforts to better understand disease mechanisms. The calcium channel blocker flunarizine improves motor function in *Smn*-deficient mice and can help uncover neuroprotective pathways. Murine motor neuron-like NSC34 cells were used to study the molecular cell-autonomous mechanism. Following RNA and protein extraction, RT-qPCR and immunodetection experiments were performed. The relationship between flunarizine mRNA targets and RNA-binding protein GEMIN5 was explored by RNA-immunoprecipitation. Flunarizine increases demethylase *Kdm6b* transcripts across cell cultures and mouse models. It causes, in NSC34 cells, a temporal expression of GEMIN5 and KDM6B. GEMIN5 binds to flunarizine-modulated mRNAs, including *Kdm6b* transcripts. *Gemin5* depletion reduces *Kdm6b* mRNA and protein levels and hampers responses to flunarizine, including neurite extension in NSC34 cells. Moreover, flunarizine increases the axonal extension of motor neurons derived from SMA patient-induced pluripotent stem cells. Finally, immunofluorescence studies of spinal cord motor neurons in *Smn*-deficient mice reveal that flunarizine modulates the expression of KDM6B and its target, the motor neuron-specific transcription factor HB9, driving motor neuron maturation. Our study reveals GEMIN5 regulates *Kdm6b* expression with implications for motor neuron diseases and therapy.

## 1. Introduction

The dysregulated assembly of ribonucleoprotein (RNP) complexes frequently leads to diseases. This is illustrated with the infantile neurodegenerative disease spinal muscular atrophy (SMA). Mutations within the *survival motor neuron 1* (SMN1) gene are responsible for SMA. The disease is characterized by the degeneration of spinal cord motor neurons and consequent skeletal muscle atrophy [1,2]. SMA severity correlates with the degree of SMN protein deficiency [3]. There is a copy gene SMN2, which produces predominantly exon 7-skipped mRNAs (SMNΔ7), resulting in insufficient levels of a fully functional SMN protein [4,5]. Thus, SMA patients suffer from SMN deficiency because they are left with SMN2 copies as the only source of gene products [6]. The search for corrections of the SMN deficit has led to the development of three innovative therapeutic interventions, significantly improving patient survival but with variable recoveries of motor function [7,8,9]. The approved therapies restore SMN protein levels. They are: nusinersen, an anti-sens oligonucleotide designed to favor exon7 inclusion in SMN2 transcripts; Zolgensma, a gene therapy that uses the adeno-associated virus 9 (AAV-9) to deliver SMN1-complementary DNA; and ridisplam, a small molecule that increases exon7 inclusion in SMN2 transcripts [10,11,12]. Nevertheless, these therapies, while impactful, have not achieved a definitive cure [9,10]. Enhanced comprehension of disease mechanisms and imperatives to explore SMN-independent combinatory therapies are essential for advancing our approach to addressing SMA and motor neuron cell death pathways.

SMN is an indispensable ubiquitously expressed protein [13,14]. It plays essential roles in RNA metabolism. The SMN protein forms a multiprotein complex featuring GEMIN2-8 and STRAP (also known as UNRIP), a complex the presence of which also diminishes under pathological conditions [15]. Its best-known function is to assemble ribonucleoprotein (RNP) complexes, including the spliceosomal small nuclear (sn)RNPs, which are major components of the spliceosome [16,17], and messenger (m)RNPs [18]. SMN is localized in both the cytoplasm and nucleus, with a notable concentration in nuclear condensates known as Cajal bodies [19]. These bodies serve as sensors of the cellular transcriptional activity [20] integrating snRNP production in the biosynthetic activity of cells [21]. Moreover, the reduced presence of SMN in CBs is a characteristic feature of motor neuron diseases such as amyotrophic lateral sclerosis (ALS) and SMA [22]. To delve deeper into their role in pathogenesis, we conducted molecular screening to identify compounds capable of recruiting SMN to CBs without changes in steady-state protein levels. The small-molecule flunarizine emerged as a positive hit in the SMA context [23]. Flunarizine is known as a calcium blocker used to treat neurological illnesses, such as vertigo, migraines, and epilepsy, and lately has been discovered as a splicing modulator of specific transcript subsets in cancer cells [24]. Motor neurons are highly vulnerable to intracellular calcium overload, owing to their low calcium buffering capacity [25]. Moreover, splicing defects were found in SMA mouse models [26,27]. Therefore, flunarizine was administered to a severe SMN-deficient mouse model, resulting in lifespan extension and preservation of spinal cord motor neurons [23]. However, details of underlying mechanisms remain largely unclear. These observations emphasize the importance of unraveling such mechanisms to uncover regulations of signaling pathways linked to RNA metabolism.

In our investigations involving SMA patient fibroblasts, flunarizine modulated the protein levels of SMN-complex components GEMINS 2–4 and accumulated GEMIN5 in enlarged nuclear sub-domains [28]. These findings raise speculation regarding the potential role of GEMIN5 in the pharmacological effects of flunarizine. Transcriptomic analysis of treated SMA fibroblasts identified around 200 genes, including 2 genes associated with cell maturation, namely the *lysine demethylase 6b* (*Kdm6b*) and the *folliculin interacting protein 1* (*Fnip1*) genes (Table S1). KDM6B is a ubiquitous protein that mediates the removal of trimethylated histone H3 lysine 27 (H3K27me3) that is tightly linked with transcriptional repression [29]. This mark can inhibit the transcription of SMN promoters [30]. The loss of H3K27me3 de-repressed genes, encoding transcription factors related to development (particularly the nervous and cardiac systems) and cell differentiation [31]. Moreover, KDM6B has been recently shown to regulate motor neuron differentiation and subtype diversification [32]. It acts as a coactivator of the transcriptional complex Isl1-Lhx3, promoting the expression of target genes such as *Mnx1*, encoding the motor neuron-specific transcription factor homeobox 9 (HB9), with an essential role in the consolidation of motor neuron cell fate [33]. FNIP1 is a co-chaperone of heat-shock protein 90 (HSP90) [34]. FNIP1 finely modulates HSP90 capacity to activate its diverse array of protein clients. Interestingly, NUFIP (nuclear FMR1 interacting protein 1), HSP90, and a co-chaperone complex, R2TP, bind the SMN complex and assist in the formation of U4 snRNA-containing RNP particles [35]. GEMIN5 directly binds snRNAs, including U4 [36,37], and might form a platform for HSP90 to chaperone U4 in snRNP assembly. Moreover, HSP90 is essential for SMN2 exon7 splicing under hyperthermic conditions [38], regulating the reductive stress response [39] and intracellular calcium levels [40]. Furthermore, FNIP1 modulates muscle fiber type specification, resistance to fatigue, and susceptibility to neuromuscular diseases [41].

The significant link between the two genes, *Kdm6b* and *Fnip1,* and flunarizine underscores the importance of exploring their role in neuroprotection. In this study, we first show that flunarizine-mediated increases in *Kdm6b* and *Fnip1* transcripts in the spinal cords of neonatal mice are independent of SMN protein levels. Thus, flunarizine-treated murine motor neuron-like NSC34 cells represent an optimal scenario by which to explore the cell-autonomous chronology of modulations in transcript and protein levels. We show that flunarizine induces a transient increase in GEMIN5 protein levels after 1 h of treatment, followed by gradual increases in *Kdm6b* and *Fnip1* mRNA levels. Interestingly, GEMIN5 binds *Kdm6b* and *Fnip1* transcripts. Moreover, depletion experiments uncovered the role of GEMIN5 in the early effects of flunarizine on these transcript levels. Among the RNA targets of GEMIN5 identified here, *Fnip1* transcripts were the most enriched targets. However, the drug did not modulate FNIP1 protein levels in NSC34 cells, whereas KDM6B levels were significantly increased, along with its downstream target, the generic motor neuron marker *Mnx1*, encoding HB9. Furthermore, flunarizine enhanced the expression of KDM6B and HB9 in the motor neurons of spinal cords from the control and SMN-deficient mice. In summary, our study identified GEMIN5 as a regulator of KDM6B involved in transcriptional networks controlling motor neuron maturation.

## 2. Results

### 2.1. SMN-Complex Component Gemin5 Expression Levels Are Modulated by Flunarizine

**Figure 1:**
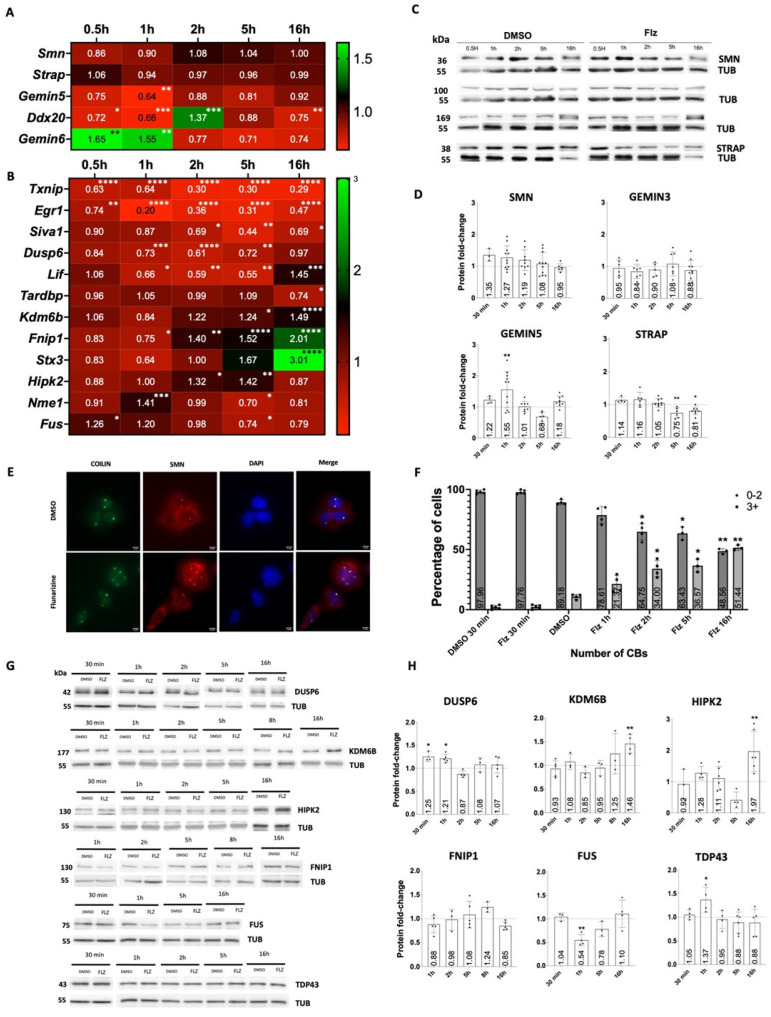
SMN-complex component GEMIN5 and neuro-developemental genes are modulated by flunarizine. (**A**) Heatmap representation of RT-qPCR analyses of genes encoding SMN-complex components in murine NSC34 cells treated with flunarizine and normalized to DMSO (diluent, arbitrary value of 1) at different time points. Two internal control genes (30 min, 1 h, 2 h: *Rpl13a*, *Hmbs*; 5 h, 16 h: *Sdha*, *Hprt1*) were used for normalization. (3 ≤ *n* ≤ 8 independent experiments, one-way Anova followed by Dunnet’s multiple *t*-test, * *p* < 0.05, ** *p* < 0.01, *** *p* < 0.001, **** *p* < 0.0001). (**B**) Heatmap of normalized expression levels of candidate RNA targets upon flunarizine treatment as detected by RT-qPCR. (**C**) Immunoblot analysis of SMN complex components in NSC34 cells treated with flunarizine compared to DMSO using α-tubulin as a loading control. (**D**) Quantification of immunoblots shown in (**C**) from 3 to 12 independent experiments. Data are represented as mean ± SEM. (one-way Anova followed by Fisher’s LSD test, * *p* < 0.05, ** *p* < 0.01). (**E**) Fluorescence images of immunostaining experiments for NSC34 cells treated with either DMSO or flunarizine for 2 h and stained with anti-coilin (green), anti-SMN antibodies (red), and bis-benzimide (blue). The microscope was focused on nuclear foci. Scale bar: 10 μm. (**F**) Analysis of Cajal body number in NSC34 cells treated with flunarizine at different time points and compared to DMSO-treated cells. Error bars indicate the SD (four independent experiments, Turkey’s multiple comparison test, * *p* < 0.05, ** *p* < 0.01). (**G**) Immunoblot analysis of flunarizine targets in NSC34 cells treated at different time points. (**H**) Quantification of immunoblots shown in (**G**) from four independent experiments.

To investigate the cell-autonomous effects of flunarizine, the murine motor neuron-like NSC34 cell line, a hybrid line produced by the fusion of neuroblastoma and motor-neuron-enriched cells from embryonic (E12–E14) spinal cords [42] was used. E12 is an early stage of motor neuron differentiation, with post-mitotic motor neurons being generated between E9 and E11 [43]. The time course of changes in the RNA and protein levels of SMN-complex components and potential targets after flunarizine treatment is shown in Figure 1A–D. The qPCR primers were designed following MIQE guidelines (Table S2). SMN RNA levels were not significantly changed as expected, whereas *Ddx20* (encoding GEMIN3) and *Gemin5* mRNA levels were transiently reduced after 1 h of treatment with *Gemin6* mRNA levels transiently increased. We previously showed flunarizine to induce a transient increase in the protein levels of SMN-complex components GEMIN2 to 4 in SMA patient fibroblasts [28]. Using specific antibodies (Table S3), a transient increase in GEMIN5 protein levels was observed after 1 h of treatment in NSC34 cells, whereas no increases were shown for the other components, except for a reduction in STRAP from 5 to 16-h treatment (uncropped images are in the Supplemental Information File).

We previously showed in SMA patient fibroblasts and the motor neurons of SMN-deficient mice that flunarizine increases SMN localization in nuclear condensates, named Cajal bodies [23]. The number of Cajal bodies is indicative of transcriptional activity [44,45] and SMN is involved in their formation [46,47,48]. Moreover, *STRAP* depletion enhanced the formation of SMN-positive Cajal bodies in cancer HeLa cells [49]. Flunarizine-treated NSC34 cells were subjected to indirect immunofluorescence experiments with the Cajal body marker coilin to evaluate the Cajal body number at different time points (Figure 1E,F). Flunarizine enhanced the proportion of cells, with 3 to 4 Cajal bodies after 2 h of treatment. This is an optimal Cajal body number for splicing snRNP assembly, on which transcriptional activity depends [44]. SMN localization in Cajal bodies was also confirmed (Figure 1E). These results indicated that Cajal body formation was influenced by flunarizine, supporting the notion of transcriptional demand.

### 2.2. Expression of Key Motor Neuron Genes Is Modulated by Flunarizine

We previously conducted a genome-wide RNA sequencing study using SMA patient fibroblasts treated with flunarizine [28]. Several genes were noteworthy in the context of SMA (Table S1). Genes regulated by the drug were associated with stress responses (*Txnip*, *Egr1*, *Siva1*), cell survival (*Lif*, *Hipk2*, *Stx3*, *Il6*, *Gdnf*, *Gdf15*, *Agrn*), cell maturation (*Kdm6b*, *Fnip1*), and RNA metabolism (*Dusp6*, *Nme1*, *Cbx8*, *Bcorl1*, *Cwc27*). Several of them have implications in motor neuron physiology. Reducing HIPK2 activity improves phenotypes in mouse models of motor neuron disease ALS [50] and IL6 levels [51], and the splicing of *Fnip1* is altered in ALS conditions [52]. LIF, GDNF, and GDF15 are potent survival factors for motor neurons [53,54,55,56,57], AGRIN (encoded by *Agrn*) is secreted by motor neurons at neuromuscular junctions [58], and RNA metabolism is altered in SMA and ALS patients associated with *Fus* or *Tardbp* mutations [22]. Gene expression was analyzed by RT-qPCR to determine changes over time in flunarizine-treated NSC34 cells (Figure 1B and Figure S1). The qPCR primers were designed specifically for one region present in all mRNA isoforms or for a given isoform (Table S2). The expression of *Txnip* and *Egr1* mRNAs was rapidly reduced upon treatment, whereas pro-apoptotic gene *Siva1* expression was reduced during 2 to 16 h treatments. Interestingly, *Kdm6b*, *Fnip1*, and *Stx3* mRNA levels gradually increased with use of the drug. We also observed transient changes for *Dusp6*, *Hipk2*, and exon inclusion in *Kdm6b* and *Strap* mRNAs (Figure S2). Finally, *Gdnf*, *Bcorl1*, and *Cwc27* genes did not undergo changes in NSC34 cells (Figure S1). Although, for most genes, there is a low correlation between transcript and protein levels, these transcriptional results led us to ask whether they could be reflective of changes in protein levels (Figure 1G,H). Immunoblot experiments confirmed the transient increase in DUSP6 (MAPK/ERK inhibitor) levels at early time points. We also observed a marked increase in protein levels for KDM6B and HIPK2 after 16 h treatment. These findings showed that flunarizine transcriptionally activates genes implicated in cell-type maturation, such as *Fnip1* and *Kdm6b*.

### 2.3. Flunarizine-Modulated Transcripts Are Immunoprecipitated by GEMIN5

**Figure 2:**
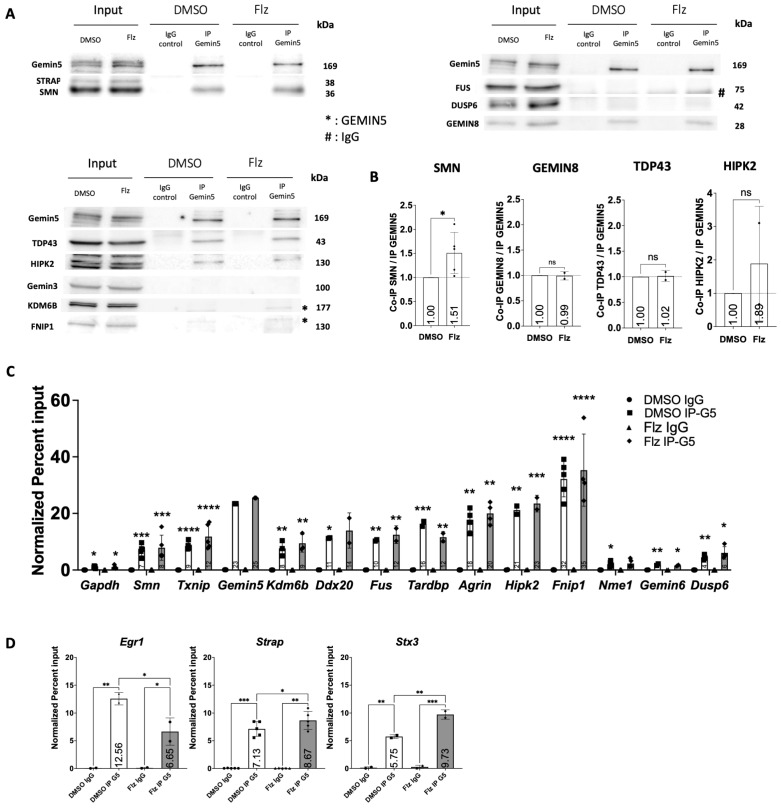
GEMIN5 associates flunarizine-modulated transcripts. (**A**) Immunoblot analyses of proteins co-immunoprecipitated with GEMIN5-RNA complexes using specific anti-GEMIN5 antibodies and cell extracts of NSC34 treated with DMSO or flunarizine (Flz) and inputs (10%). (**B**) Quantification of the ratio of protein over GEMIN5 in immunoprecipitated complexes from panel (**A**), two to five independent experiments. (**C**) RT-qPCR analysis of RNA molecules associated with GEMIN5-RNA complexes compared to non-specific IgG using DMSO or flunarizine (Flz)-treated NSC34 cells. Percent inputs were calculated according to the constant volume method and normalized to the percent input value of *Gemin5* transcripts. *Gapdh* transcripts were used as the negative control. Error bars indicate the SD (2 ≤ *n* ≤ 5 independent experiments, one-way Anova followed by Turkey’s multiple comparison test, « ns » not significant (*p* > 0.05), * *p* < 0.05, ** *p* < 0.01, *** *p* < 0.001, **** *p*< 0.0001). (**D**) RT-qPCR analysis of RNA molecules associated with GEMIN5-RNA complexes modulated by flunarizine. Quantification was as in panel (**C**).

Given that ENCODE cross-linking immunoprecipitation (CLIP)-based studies with human lymphoblastoid cells have revealed genome-wide mRNA targets of GEMIN5 (ENCSR238CLX), we aimed to determine if flunarizine-modulated mRNAs could be bound to GEMIN5 in NSC34 cells. RNA-immunoprecipitation (RIP) was performed against GEMIN5 during 2 h flunarizine treatments followed by RT-qPCR on immunopurified RNAs together with input samples to define total RNA levels. RIP was carried out under stringent non-crosslinked conditions and compared to the IgG-negative control antibodies. The specificity of GEMIN5 immunoprecipitation was validated through immunoblotting (Figure 2A). Immunopurified complexes contained GEMIN5, SMN, and GEMIN8, but neither GEMIN3 nor STRAP, suggesting a separation of SMN-complex components, as previously reported [37]. GEMIN5 also co-purified TDP-43 (encoded by *Tardbp*) and HIPK2, whereas FUS and DUSP6 were not purified. Moreover, GEMIN5 RIP showed an enrichment of SMN protein in flunarizine-treated cells (Figure 2A,B).

To measure immunoprecipitated RNAs, we applied the percent input values of the constant volume method to analyze nucleic acid-binding to proteins [59]. Previous reports showed *Smn*, *Txnip*, and *Gemin5* transcripts to be bound to GEMIN5 [60,61,62]. We used positive (*Gemin5* mRNA, percent inputs of ≈25%) and negative control RNAs (*Gapdh* mRNA, percent inputs of ≈1%) to validate our experimental approach (Figure 2C). All transcripts had a percent input of ≈0.025% with the IgG negative control. To account for variations in immunoprecipitated GEMIN5 levels among experiments, an average of *Gemin5* transcript percent input values was calculated and used to normalize the percent input values of the other mRNAs. We revealed that *Kdm6b* transcripts had similar normalized percent input values to *Smn* transcripts in both DMSO and flunarizine samples. *Ddx20* and *Fus* transcripts behaved like *Txnip* transcripts. *Tardbp*, *Hipk2*, and *Agrn* transcripts were similar to *Gemin5* mRNAs, and *Fnip1* transcripts had the highest normalized percent input values ever reported for GEMIN5 RNA targets, with a value of ≈32%. We also observed that *Nme1* and *Gemin6* transcripts had similar normalized percent input values as the negative control. Finally, flunarizine modulated the normalized percent input values of *Strap*, *Egr1*, and *Stx3* transcripts in NSC34 cells (Figure 2D). These results indicate that *Kdm6b* and *Fnip1* transcripts can be bound to GEMIN5 and that flunarizine can influence RNA-protein interactions.

### 2.4. Gemin5 Depletion Mimics the Early Time Point of Flunarizine Treatment

**Figure 3:**
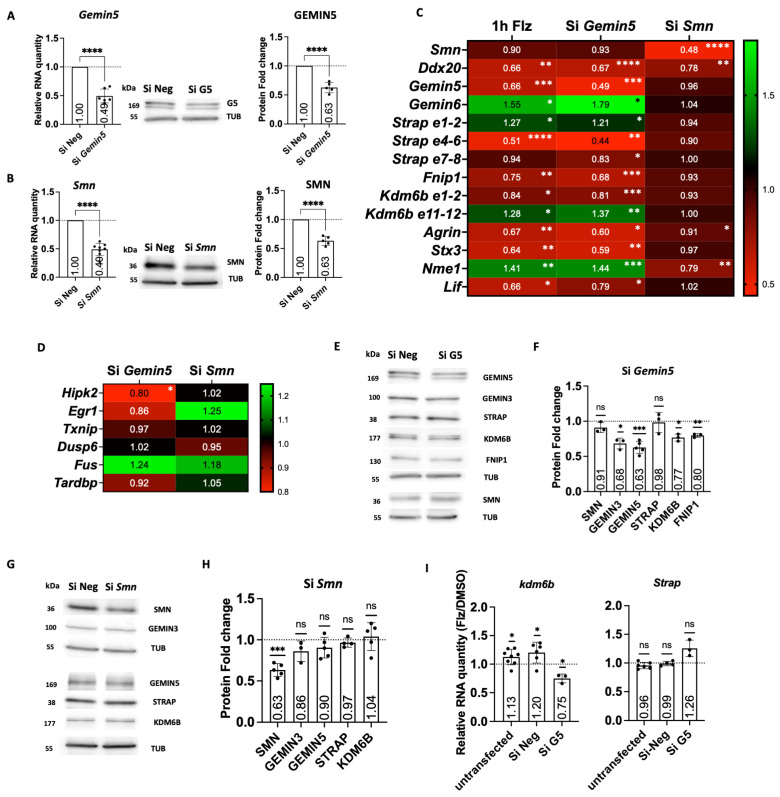
*Gemin5* depletion mimics the early time point of flunarizine treatment. (**A**) RT-qPCR and immunoblot analyses of *Gemin5* expression in si*Gemin5*-treated NSC34 cells. (**B**) RT-qPCR and immunoblot analyses of *Smn* expression in si*Smn*-treated NSC34 cells. (**C**) Heatmap of normalized expression levels for SMN-complex components in NSC34 cells after the indicated treatments as detected by RT-qPCR. (**D**) Heatmap of gene expression in NSC34 cells after the indicated treatments as detected by RT-qPCR. (**E**) Immunoblot analysis of protein expression in si*Gemin5*-treated NSC34 cells using α-tubulin (tub) as a loading control. (**F**) Quantification of immunoblot shown in (**E**) from three to five independent experiments. DMSO was given an arbitrary value of 1. Error bars indicate the SD (3 ≤ *n* ≤ 8 independent experiments, one sample *t*-test, « ns » not significant (*p* > 0.05), * *p* < 0.05, ** *p* < 0.01, *** *p* < 0.001). (**G**) Immunoblot analysis of protein expression in NSC34 cells treated with si*Smn*. (**H**) Quantification of immunoblot shown in (**G**), three to five independent experiments. (**I**) RT-qPCR analysis of *Kdm6b* and *Strap* transcripts upon a 5-h flunarizine treatment of *Gemin5*-depleted NSC34 cells. Internal control genes (*Actb* and *Hmbs*) were used for normalization. DMSO was given an arbitrary value of 1. (3 ≤ *n* ≤ 8 independent experiments, one sample *t*-test, « ns » not significant (*p* > 0.05), * *p* < 0.05, ** < 0.01, *** *p* < 0.001, **** *p* < 0.0001).

To further understand how GEMIN5 was implicated in flunarizine mechanisms of action, we examined the effects of *Gemin5* (si*Gemin5*) or *Smn* depletion (si*Smn*) on the expression of flunarizine targets and compared mRNA levels with those after 1 h treatment. Depletions were validated at both RNA and protein levels using RT-qPCR and immunoblotting, respectively (Figure 3A,B). Both siRNAs reached 40 to 50% reduction at mRNA and protein levels. Importantly, *Gemin5* depletion did not reduce *Smn* mRNA or protein levels, and *Smn* depletion did not reduce *Gemin5* transcripts or protein levels in our experimental conditions using NSC34 cells (Figure 3C,E–H). However, *Ddx20* mRNA levels were similarly reduced by both siRNAs (Figure 3C). Effects on *Ddx20*, *Gemin6*, *Strap*, *Kdm6b*, *Fnip1*, *Agrn*, *Stx3*, *Nme1*, and *Lif* mRNA levels were similar between si*Gemin5* and 1 h drug treatments, while *Hipk2* mRNA levels were reduced (Figure 3C,D). The mRNA levels of *Egr1*, *Txnip*, *Dusp6*, *Fus*, and *tardbp* were unchanged by either *Gemin5* or *Smn* depletion (Figure 3D). Notably, GEMIN3 and KDM6B protein levels were reduced by *Gemin5* depletion but not by *Smn* depletion, although both siRNAs reduced *Ddx20* mRNA levels, indicating that GEMIN5 but not SMN can stabilize GEMIN3 at the protein level (Figure 3E–H). Moreover, *Gemin5* depletion prevented flunarizine from working on *Kdm6b* transcripts (Figure 3I). These results suggest that GEMIN5 is implicated in the mode of action of flunarizine at early time points, uncovering a molecular link between GEMIN5 and *Kdm6b* expression.

### 2.5. Neurite Outgrowth Is Promoted in Cell Cultures by Flunarizine

**Figure 4:**
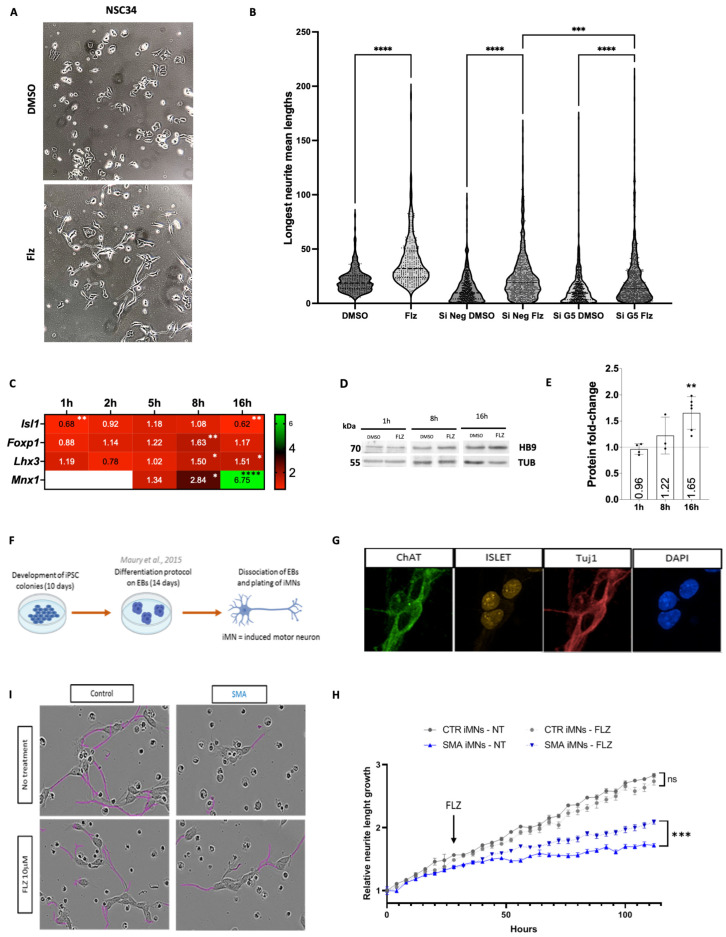
Flunarizine promotes neurite outgrowth. (**A**) Micrographs for neurite outgrowth after 16-h DMSO and flunarizine treatments in NSC34 cells. (**B**) Length of the longest neurite of each individual NSC34 cell treated with DMSO or flunarizine (Flz) with or without siRNA *Gemin5* (siG5) or negative control (si*Neg*) transfections. Three independent experiments, unpaired *t*-test, **** *p* < 0.0001. (**C**) Heatmap representation for gene expression of KDM6B downstream targets in NSC34 cells treated with flunarizine at different incubation time points as detected by RT-qPCR. Internal control genes (1 h, 2 h: *Rpl13a*, *Hmbs*; 8 h-5 h-16 h: *Sdha*, *Hprt1*) were used for normalization. Error bars indicate the SD (3 ≤ *n* ≤ 8 independent experiments, one-way Anova followed by Fisher’s LSD test, « ns » not significant (*p* > 0.05), * *p* < 0.05, ** *p* < 0.01, *** *p* < 0.001, **** *p* < 0.0001). (**D**) Immunoblot analysis of HB9 encoded by *Mnx1* upon flunarizine treatment of NSC34 cells at different time points and compared to DMSO treatment. The α-tubulin was a loading control. (**E**) Quantification of immunoblots in panel (D). An arbitrary value of 1 is given to DMSO. (**F**) Differentiation protocol adapted from Maury et al. [65] to differentiate iPSCs into induced motor neurons. (**G**) Immunofluorescence image of differentiated motor neurons stained for Tuj1, ChAT, and ISLET (motor neuron markers). (**H**) Motor neurons imaged at D14 after 72 h of treatment or not with flunarizine (FLZ). Images were taken in phase contrast on IncuCyte^®^ and neurite detection (in purple) was automated with the IncuCyte software. (**I**) Neurite length growth was quantified from plating (H0) and throughout FLZ treatment. Neurite length is relative to the length at H0.

We unexpectedly observed that neurite length increased in NSC34 cells with 16-h flunarizine treatment (Figure 4A). SMN-deficiency causes defects in neurite outgrowth [63] and enhancement of neurite outgrowth might improve SMA conditions. Therefore, the length of the longest neurite was measured using tubulin beta III (also known as the clone name TUJ1) for immunofluorescent labeling (Figure 4B). Flunarizine significantly increased the length of the longest neurite compared to DMSO treatment. It was also observed that cell density was increasing with DMSO compared to flunarizine treatment, suggesting that flunarizine might reduce proliferation and favor cell maturation. Moreover, *Gemin5* depletion prevented flunarizine from working on neurite extension (Figure 4B). Given the role of KDM6B in motor neuron maturation [32] and its increase upon flunarizine treatment as shown in Figure 1, we also explored gene expression of its interactors and downstream targets upon flunarizine treatment. KDM6B interacts with the transcription factor complex Isl1-Lhx3, driving motor neuron diversification and *Mnx1* expression [32]. *Isl1*, *Lhx3*, *Foxp1*, and *Mnx1* genes encode transcription factors that are skeletal motor neuron hallmarks [64]. The homeobox HB9 (encoded by *Mnx1*) is required for the consolidation of motor neuron identity [33]. We observed in NSC34 cells an increase in *Lhx3* mRNA levels from 8 to 16-h treatment, whereas *Isl1* transcripts were decreased at 16-h treatment (Figure 4C). *Foxp1* mRNA levels were transiently increased at 8-h treatment only. Importantly, *Mnx1* mRNA levels were the most upregulated, with a ≈7-fold increase at 16-h treatment that was also associated with increased HB9 protein levels (Figure 4D,E). These data indicate that up-regulation of *Kdm6b* and *Mnx1* is a significant downstream event of flunarizine treatment.

Previous studies showed that iPSCs-derived motor neurons from SMA patients exhibit defective neurite outgrowth [66,67]. In the present study, induced iPSC-derived motor neurons from control and SMA patients displayed characteristic neural morphologies and expressed the motor neuron hallmarks, namely choline acetyltransferase (ChAT), ISL1, and TUJ1 (Figure 4F,G). To evaluate the effects of flunarizine on neurite growth, neurite elongation was monitored (Figure 4H,I). An arbitrary unit of 1 was given at the starting point, and flunarizine was added 25 h later. Neurite elongation was lower in SMA iPSC-derived motor neurons than in controls. Moreover, flunarizine significantly increased elongation in SMA iPSC-derived motor neurons. These results further confirmed that flunarizine acts directly on motor neuron cells in a manner independent of SMN protein levels.

### 2.6. Flunarizine Stimulates KDM6B and HB9 Expression in Motor Neurons of Smn-Deficient Mice

**Figure 5:**
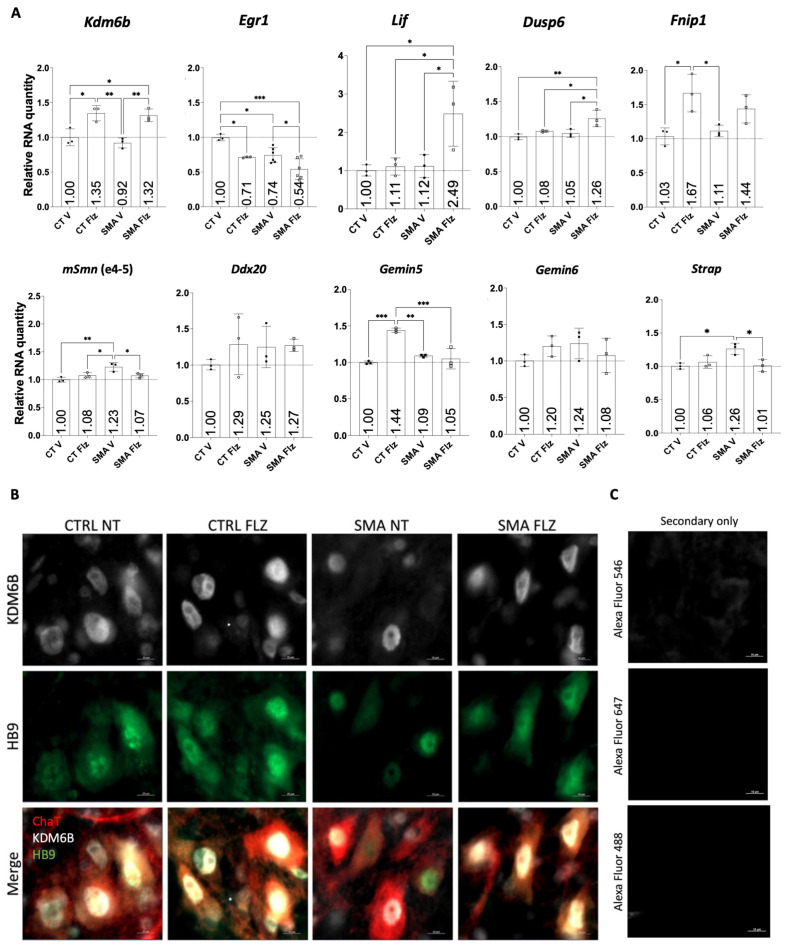
Flunarizine stimulates KDM6B and HB9 expression in motor neurons of SMN-deficient mice. (**A**) RT-qPCR analysis of candidate RNA targets and SMN-complex components in the spinal cord of vehicle (V)- or flunarizine (Flz)-treated heterozygote control (CT) and SMN-deficient mice (SMA) at postnatal day P10. Internal control genes were used for normalization (*Rpl13a*, *Actb*, *Ppia*). The control vehicle was given an arbitrary value of 1. Error bars represented the standard deviation (SD) from the mean values of triplicates from 3 ≤ *n* ≤ 6 mice per group. (One-way Anova followed by Turkey’s multiple comparisons test. « ns » not significant (*p* > 0.05), * *p* < 0.05, ** *p* < 0.01, *** *p* < 0.001). (**B**) Fluorescence images of ChAT (red), KDM6B (white), and HB9 (green) immunostaining of spinal cord sections from heterozygote control (CTRL) and SMN-deficient (SMA) mice at P5 treated or not (NT) with flunarizine (FLZ). The microscope was focused on KDM6B labeling. Scale bar 10 µm. (**C**) Immunostaining with fluorescent secondary antibodies only.

Given that flunarizine can improve neurite projection in motor neuronal cells (Figure 4) and that *Kdm6b* plays a crucial role in motor neuron maturation, we examined at post-natal age P10 the RT-qPCR mRNA levels of *Kdm6b* in the spinal cord of heterozygote (control) and SMA mutant mice treated with either vehicle (V) or flunarizine (Flz) (Figure 5A). We used the Taiwanese mouse model that carries homozygous deletion of *Smn* exon7 in addition to transgenic human *SMN2* genes [14]. The mRNA levels were also examined for *Egr1*, *Lif*, *Dusp6*, *Fnip1*, *Smn* alleles (normal and mutated), *Ddx20*, *Gemin5*, *Gemin6*, and *Strap*. *Kdm6b* mRNA levels were increased by flunarizine in controls and mutants, whereas *Egr1* mRNA levels were reduced in both groups. Moreover, flunarizine increased *Lif* and *Dusp6* whereas decreased *Strap* mRNA levels in SMA mutants. The drug increased *Gemin5* and *Fnip1* mRNA levels in control animals. Also, *Fnip1* mRNA levels exhibited a trend toward increased expression in SMA mutants with the drug. In brains, *Egr1*, *Fnip1*, and *Dusp6* mRNA levels were not significantly changed by flunarizine, whereas *Kdm6b* and *Gemin5* mRNA levels were reduced in SMA mutant brains (Figure S4). Together, we confirmed the regulation of relevant genes for motor neuron physiology by flunarizine in the spinal cord of control and *Smn*-deficient mice.

The correlation between increased mRNA and protein levels for *Kdm6b* in flunarizine-treated NSC34 cells (Figure 1H) raised the question whether the same was true in vivo when *Kdm6b* transcripts were increased in the spinal cord of flunarizine-treated control and *Smn*-deficient mice. To ask the question, we performed immunohistochemical experiments in the lumbar spinal cord of control and SMN-deficient mice treated or not with flunarizine at age P5 (Figure 5B). We focused on an early symptomatic stage preceding motoneuron death [68]. The spinal cord sections were co-stained for ChAT, KDM6B, and HB9 using specific antibodies (Table S3). We found that ChAT-positive cells displayed KDM6B and HB9 co-labelling in the spinal cord of the untreated control, whereas fewer ChAT-positive cells were co-labeled in untreated *Smn*-deficient mice. Remarkably, flunarizine enhanced KDM6B and HB9 co-labelling in ChAT-positive cells in *Smn*-deficient mice. These results support the involvement of KDM6B and HB9 in neuroprotection of motoneurons by flunarizine.

## 3. Discussion

The need to progress beyond gene discovery in rare motor neuron disorders to disease mechanisms is highlighted by clinical observations that actual therapies are not cures [7,9]. In this study, we looked at mechanisms of action related to RNA metabolism and transcription regulation for flunarizine in neuroprotection. Our previous report showed that flunarizine improves the phenotype of SMN-deficient mice [23]. We address here the cell-autonomous issue of neuroprotection by investigating flunarizine impact on potential targets [28]. We show a temporal role of GEMIN5 on the mRNA and protein levels of flunarizine targets. The striking ability of flunarizine to modulate motor neuron-specific transcription factors suggests a strong effect on motor neuron maturation during neonatal development.

To get to these conclusions, we use the murine motor neuron-like NSC34 cell line, Gemin5 RNA-immunoprecipitation, and evaluate at different time points the expression of flunarizine RNA targets and key transcription factors of motor neurons. Four lines of evidence implicate GEMIN5 in the mode of action of flunarizine. First, the drug transiently increases *Gemin5* protein levels at 1-h of treatment (Figure 1C). Second, it modulates the binding of GEMIN5 to flunarizine mRNA targets (Figure 2C). Third, *Gemin5* depletion with a protein reduction of ≈40% is sufficient to mimic the effects observed on flunarizine-modulated transcripts at 1-h treatment, whereas *Smn* depletion is not effective on those transcripts (Figure 3C). Forth, *Gemin5* depletion hampers the effects of flunarizine on *Kdm6b* transcripts (Figure 3I). Moreover, we demonstrate a temporal activation of cell-autonomous targets in response to flunarizine (Figure 1). Treatment with the drug leads to a rapid increase in protein levels of DUSP6, a MAPK/ERK inhibitor, within the first hour that returns to initial protein levels at the 2-h time point. The accumulation of DUSP6 is probably due to stabilization because it happens without an increase in *Dusp6* mRNA levels. It correlates with a marked reduction of *Egr1* transcripts that is irreversible over the 16–24-h period of our study. This reduction of *Egr1* mRNA levels was also seen in spinal cord of control and SMA mice (Figure 5A). At 30 min treatment of NSC34 cells, the modest and transient increase of SMN protein levels is consistent with findings that pharmacological inhibition of ERK can increase *SMN2* mRNA levels in SMN-deficient mice [69]. All together, these observations strongly suggest that ERK activity is decreased at first. Other studies have shown that ERK can also regulate *Dusp6* expression [70]. Indeed, we observe a reduction in *Dusp6* mRNA levels between 1- to 5-h treatments, suggesting that DUSP6 inhibits ERK, reducing *Dusp6* mRNA levels that can be restored later on by a feedback loop. Indeed, *Dusp6* mRNA levels are back to initial levels at 16-h treatment, suggesting that ERK has returned to control activity.

Flunarizine-modulated transcripts are either increased or reduced at 2-h, indicating a transcriptional switch around this time point. It is also at 2-h treatment that GEMIN5-RNA complexes show an increase of association with SMN protein by flunarizine, whereas TDP-43 association is not enhanced. The RNA/DNA-binding factor TDP-43 is a ubiquitously expressed nuclear protein involved in pre-mRNA maturation [71]. In TDP-43 proteinopathies, the loss of nuclear TDP-43 correlates with dysregulation of mRNA targets and TDP-43 accumulation in cytoplasmic aggregates of affected neurons [72,73]. A previous study of splicing events controlled by TDP-43 in the context of the motor neuron disease ALS identified a downregulated exon in *Fnip1* transcripts [52]. This suggests that GEMIN5 and TDP-43 association might depend on the presence of *Fnip1* transcripts. Interestingly, *Fnip1* and *Kdm6b* transcript levels are also upregulated by the drug at 2-h and remain high over our timeline. Given that the Gemin5-associated fraction of *Fnip1* and *Kdm6b* transcripts is unchanged by the drug at 2 h but mRNA levels are upregulated, it indicates that more mRNA molecules are associated with GEMIN5 with flunarizine. This association might negatively modulate protein synthesis since a modest and transient increase in *Fnip1* protein levels is detected and a temporally progressive increase in *Kdm6b* protein levels begins later on, being significant at 16 h treatment.

The SMN complex is involved in the assembly of various RNA-protein particles, including snRNPs and mRNPs [15,74]. GEMIN5 plays important roles in addition to operating as a component of the SMN complex. GEMIN5 was identified as a ribosome-binding protein and as a negative regulator of protein synthesis [75]. Other studies showed that GEMIN5 can promote translation of selective mRNAs [60,61,62]. It binds its own mRNA, providing a feedback loop to regulate its protein levels [61]. It also binds *SMN* mRNA, increasing its translation [60] and to the 5′ UTR of viral RNAs, regulating viral protein synthesis [76]. In addition, it was identified as a factor bound to viral internal ribosomal entry site (IRES) elements modulating IRES-dependent translation of targets such as *Txnip* mRNAs [62,77]. It is because of this interaction between GEMIN5 and *Txnip* transcripts together with the transient increase of *Gemin5* at the protein level with 1-h flunarizine treatment that we hypothesized a role for GEMIN5 in the mechanisms of action of the drug. Moreover, human mutations in the SMN or GEMIN5 gene cause different disease phenotypes. Indeed, SMN gene alterations lead to a motor neuron disease, whereas GEMIN5 mutations cause neurodevelopmental diseases [78,79,80,81,82]. An elegant study of transcriptional profiling of murine motor neuron maturation revealed that *Smn* and *Gemin5* genes are highly expressed during embryonic age and at lower levels after birth, *Gemin5* mRNA levels remaining the highest [64]. We show here that *Gemin5* depletion causes a clear reduction of *Kdm6b* mRNA and protein levels. Given that loss-of-function of the *Kdm6b* gene has also been associated with neurodevelopment disorders [83], it is tempting to speculate that dysregulated epigenetic marks might contribute to the phenotype of pathogenic *GEMIN5* variants [78,84]. In another study, GEMIN5 was shown to bind histone mRNAs and to regulate their translation using histone mRNA reporters [85]. *GEMIN5* mutations could therefore perturb histone marks and, consequently, the progression of gene programs during embryonic and postnatal development, triggering a neurodevelopmental disorder.

KDM6B can remove epigenetic marks important for tissue development to proceed through gene programs of differentiation and maturation. A sophisticated transcriptional regulatory network coordinates motor neuron identity and subtype diversification. The key players of the network are Isl1/2, Lhx3/4, Mnx1, and Foxp1. The Isl1-Lhx3 complex activates a series of motor neuron-specific genes [86,87,88,89], including *Mnx1*, which consolidates the motor neuron identity [33]. Indeed, *Mnx1* maintains Isl1 and Lhx3 expression and down-regulates interneuron fate [90]. Motor neurons of newborn mice further mature to various subtypes. Lhx3 is reduced in all motor neurons except for the medial motor column (MMC), whereas Isl1 expression persists in many subtypes; the maintenance of the Isl1-Lhx3 complex in MMC activates genes involved in terminal differentiation and axon pathfindings [86,87,88,91]. *Kdm6b* acts as a crucial co-activator of the Isl1-Lhx3 complex [32]. In embryos, *Kdm6b* promotes the generation of MMC and hypaxial motor column (HMC) and suppresses lateral motor column (LMC) and preganglionic motor column (PGC) formation. The continuous action of *Kdm6b* is needed to maintain MMC identity. The functional importance of KDM6B for neuroprotection was not previously explored in SMA and other motor neuron diseases. We found that *Kdm6b* at mRNA and protein levels is robustly expressed in the spinal cord during neonatal development in mice. This finding is consistent with the above-mentioned studies showing a role for *Kdm6b* in motor neuron maturation [32,64,92]. Moreover, the upregulation of *Kdm6b* mRNA and protein levels in spinal cord of control and SMA mutant mice by flunarizine indicates that it is independent of SMN protein levels. Flunarizine might be an option to complement the actual SMA therapies aiming to increase SMN protein levels. Perinatal period is the moment when motor neurons show selective vulnerability to reduced levels of SMN [93]. Indeed, MMC motor neurons are highly vulnerable to SMN deficiency, and convergent events are required for selective cell death [94]. We propose that flunarizine enhances motor neuron identity, making them more resistant to cell death. This is in agreement with synaptic improvements of motor neurons by flunarizine in SMN-deficient mice [23,95] and with a role for *Kdm6b* as a positive regulator of lifespan extension [96,97]. Our study on flunarizine mode of action provides insights into mechanisms of neuroprotection. Beyond motor neuron disease mechanisms, it reveals a role for GEMIN5 in the regulation of the epigenetic enzyme KDM6B expression and consequently, enabling the expression of KDM6B-Isl1-Lxh3 target gene HB9, which consolidates motor neuron identity.

In conclusion, our study reveals the RNA-binding protein GEMIN5 to regulate *Kdm6b* gene expression with implications for motor neuron diseases and therapy. We used a murine cell-culture model and targeted mRNA depletion to understand the function of GEMIN5 in the mode of action of flunarizine. Our study identifies flunarizine-induced *Kdm6b* transcripts as GEMIN5 mRNA targets. The drug enhances *Kdm6b* mRNA levels in the spinal cord of control and SMN-deficient mice. It also increases protein levels of KDM6B in spinal motor neurons independently of SMN levels. It seems likely that distinct events contribute to drug effects. It is possible that removal of repressive marks by KDM6B is involved while it might be independent of its enzymatic activity. Further study is needed to assess pathways upstream and downstream of KDM6B that may yield neuroprotective targets for SMA and other motor neuron diseases. Moreover, flunarizine promotes neurite outgrowth in both murine NSC34 and SMA patient iPSC-derived motor neurons. Taken together, these findings offer a better understanding of the beneficial effects of flunarizine in neurological treatments beyond motor neuron diseases.

## 4. Materials and Methods

### 4.1. Cell Cultures, Flunarizine Treatment, and Immunodetection

Murine motor neuron-like NSC34 cells were grown in TPP culture dishes at 37 °C in Dulbecco’s modified Eagle’s medium (DMEM)-Glutamax supplemented with 10% fetal bovine serum (FBS), penicillin (100 U/mL), streptomycin (100 mg/mL), and 5% CO_2_. The NSC34 cell line was kindly provided by Neil R. Cashman [42]. Flunarizine (F-8257, Sigma-Aldrich, St. Louis, MO, USA) was dissolved at 10 mg/mL in DMSO and further diluted at 10 μg/mL in medium for treatment. Cells were plated at a density of 150,000 cells/mL on 100 mm TPP petri dishes using 8 mL culture medium and treated with flunarizine (10 μg/mL) or diluent DMSO (0.1%) for different time points. Cells were washed with cold PBS, scraped off Petri dishes, centrifuged, and cellular pellets were frozen at −80 °C for immunoblotting. Cells were plated on 60-mm TPP petri dishes for immunofluorescence experiments, as previously described [57]. Briefly, cells were washed with PBS and fixed with 4% formaldehyde (F-8775, Sigma-Aldrich) in PBS, permeabilized with 0.5% Triton X-100, and immunostained using primary and secondary antibodies (Table S3). The final wash contained 0.1 mg/mL of bisbenzimide H33258 (B-1155, Sigma-Aldrich). Preparations were mounted in Vectashield or fluoromount gold mounting medium.

The control iPSC line was generated by the stem cell facility at the host institute (Institut Imagine cell line #Ctr004). The SMA iPSC line comes from the SMA Collection of CS iPSC Core Repository (West Hollywood, CA 90069, USA) from a 3-year-old male patient with an EX7-8DEL mutation on SMN1. iPSC colonies were grown in mTeSR medium in vitronectin (both StemCell)-coated dishes and were passaged by enzyme-free dissociation (ReleSR, StemCell) according to the manufacturer’s instructions. To generate motor neurons, we adapted a published protocol [65]. Briefly, on day 0, iPSC colonies were dissociated to single cells using Accutase (Life Technologies, Carlsbad, CA, USA) and seeded in ultra-low attachment 6-well plates (3 × 105 cells/well) or cell-repellent surface T25 flasks (3 × 105 cells/flask) in N2B27 medium. N2B27 medium consists of a 1:1 ratio of advanced DMEM/F12 (Life Technologies) and Neurobasal medium (Life Technologies), 1% N2 supplement (Life Technologies), 2% B27 supplement minus vitamin A (Life Technologies), 5 mM Glutamax (Life Technologies), 5 mM L-Glutamine (Sigma), 1% penicillin/streptomycin (Life Technologies), and 0.1 mM 2—ME (Life Technologies). In these conditions, iPSCs spontaneously aggregated into embryoid bodies (EBs) in suspension in N2B27 medium. To induce proper differentiation of these iPSCs into motor neurons, the medium was changed on specific days and different factors were added each time. On day 0, N2B27 medium + 10 μM ROCK inhibitor Y—27632, 3 μM CHIR99021 (TOCRIS), 0.1 μM LDN (TOCRIS), 20 μM SB431542 (TOCRIS), and 10 μM ascorbic acid (Sigma-Aldrich) were added. On day 2, the medium was changed to N2B27 + 3 μM CHIR99021, 0.1 μM LDN, 20 μM 8SB431542, and 100 nM retinoic acid (Sigma-Aldrich). On day 4, the medium was changed to N2B27 + 0.1 μM LDN, 20 μM SB431542, 100 nM retinoic acid, and 500 nM SAG (TOCRIS). On day 7, the medium was changed to N2B27 + 100 nM retinoic acid and 500 nM SAG. On day 9 and day 11, the medium was changed to N2B27 + 100 nM retinoic acid, 500 nM SAG, and 10 μM DAPT (TOCRIS). Finally, on day 14, the EBs were composed of motor neurons and were dissociated with 0.5% Trypsin (Life Technologies).

### 4.2. Neurite Tracking with IncuCyte^®^

On day 14 20,000 motor neurons were seeded in a 96-well plate previously coated with poly-d-lysine (PDL, Thermo Fischer, molecular weight 50,000–150,000, 0.1 mg/mL in Dulbecco’s phosphate buffered saline, D-PBS). The neurite growth follow-up of the motor neurons was carried out using the IncuCyte^®^ apparatus. Briefly, we took phase-contrast pictures of the motor neurons every 4 h for 5 days after seeding, with or without treatment with 10 µM flunarizine. The length of the neurites was automatically quantified using the IncuCyte^®^ NeuroTrack Analysis Software Module. To obtain the relative neurite length growth, the quantified neurite length for each time point was compared to the neurite length of the motor neurons at the start of the experiment (day 14).

### 4.3. Transfection Assays

NSC34 cells were freshly plated at a density of 200,000 cells/mL in 60 mm TPP dishes for transfection using DharmaFect transfecting reagent III (Horizon) with either *Smn*-validated siRNA (SI00200718, FlexiTube siRNA, Qiagen, Hilden, Germany) at 4.4 nM or *Gemin5*-validated siRNA (S101011108, FlexiTube siRNA, Qiagen) at 6.6 nM according to the manufacturer’s protocol. Controls were transfected with an equivalent concentration of Si Neg (1022076, Qiagen). Cells were incubated for 48 h, scraped off of Petri dishes in cold PBS, centrifuged, then frozen as pellets at −80 °C for immunoblotting analysis or scraped off in Trizol reagent (Fisher Scientific, Hampton, NH, USA), and then frozen at −80 °C for RNA extraction.

### 4.4. Immunoblotting Analysis

NSC34 cellular extracts were prepared from frozen pellets at −80 °C. The pellets were resuspended in Tris-NaCl buffer [50 mM Tris–HCl (pH 7.4), 150 mM NaCl] supplemented with an EDTA-free protease inhibitor cocktail (Roche). The protein concentrations were determined using the Bradford assay (Bio-RAD Laboratories, Hercules, CA, USA). 30–90 ug of proteins were diluted in the loading Laemmli buffer to a final concentration of 1–2 ug/uL, heated at 95 °C for 5 min, and processed for gel electrophoresis. The proteins were resolved on 10% ProSieve 50 poly-acrylamide gel (FMC Bioproducts, Rockland, ME, USA) in Tris-Tricine running buffer and transferred to PVDF membranes (Millipore). The membranes were blocked in 10% nonfat dry milk in PBS-Tween (0.05%) for 1h at room temperature and incubated with primary antibodies (Table S3) diluted in 5% nonfat dry milk for 1 h at RT or overnight at 4 °C. The membranes were washed for about 30 min in 3 changes of PBS-Tween and incubated at room temperature for 1 h with CleanBlot secondary antibodies at 1:1000 to 1:2000 conjugated to the horseradish peroxidase (HRP) enzyme. When necessary, the membranes were stripped (Restore WB stripping buffer, Thermo Scientific) and sequentially probed with other antibodies. The protein bands were visualized using chemiluminescence (Amersham ECL, GE Healthcare) with ImageQuant LAS 4000 (GE Healthcare). Intensity analysis was conducted using ImageJ software, Version 2.9.0/1.53t.

### 4.5. RNA Extraction and Expression Analysis

Total RNA was extracted from tissues and cultured NSC34 cells using Trizol Reagent (Fisher Scientific) and treated with an RQ1 RNase-free DNase (Promega). One µg of RNA was used to generate cDNA with Superscript III (Invitrogen). Quantitative real-time PCR was performed in triplicates with SYBR Green ROX mix (Thermo Scientific) on BioRad CFX384. The normalized expression levels were calculated according to the ΔΔCt method using reference genes indicated in figure legends. Primers have been designed using the free primer design tools from Eurofins (eurofinsgenomics.eu) or IDT (idtdna.com) and validated according to MIQE guidelines.

### 4.6. RNA Immunoprecipitation

The cellular extracts were prepared from frozen NSC34 pellets kept at −80 °C. The pellets were lysed in [25 mM Tris–HCl (pH 7.4), 150 mM KCl, 1% NP40, 5 mM EDTA] supplemented with [0.5 mM DTT, 40 U/mL of RNAse out (Invitrogen) and EDTA-free protease inhibitor cocktail (Roche)], passed through 26-gauge and 30-gauge needles, and clarified by centrifugation at 2600× *g* for 5 min at 4 °C. The supernatant was collected and pre-cleared with 100 µL of Dynabeads protein A (Invitrogen) for 2 h at 4 °C. Then, protein concentrations were determined using the Bradford assay (Bio-RAD Laboratories). Three ug of anti-GEMIN5 (GTX130498) or of negative control IgG (DAKO X0903) were diluted in lysis buffer and added to 100 µL of Dynabeads protein A and incubated overnight with rotation at 4 °C. Dynabeads-antibodies complexes were washed 3 times in lysis buffer, then incubated with 2500–3000 µg of cellular extracts overnight. Then, 10% of cellular extracts were kept aside to serve as inputs (INs). Dynabeads were washed three times in lysis buffer and divided in two for immunoblotting analysis and RNA extraction. One half was eluted in loading Laemmli buffer and the other half in Trizol Reagent (Fisher Scientific). The following steps adhere to the protocols for immunoblotting and RNA expression analysis by RT-qPCR. Percent inputs were calculated based on the constant volume method using the following equation: % input = 2^((Cq(IN)−Log2(DF))−Cq(IP))^ ∗ 100, DF being the dilution factor. To account for inter-experiment differences, percent inputs were normalized to the percent input of *Gemin5* mRNA, a known strong positive control.

### 4.7. Animal Procedure and Spinal Cord Tissue Experiment

The severe Taiwanese SMA mouse model (*Smn*^ko/ko^; huSMN2^tg/0^) and corresponding control heterozygous (*Smn*^ko/wt^; SMN2^tg/0^) mice were produced in the same litter. Indeed, transgenic mild SMA-like males (*Smn*^ko/ko^; huSMN2^+/+^ four copies, FVB.Cg-*Smn1t^m1Hung^* Tg(SMN2)2Hung/J, strain #005058) [14] were obtained from Jackson Laboratory and crossed with heterozygous *Smn* knock-out females (*Smn*^ko/+^), to generate 50% of severe Taiwanese SMA mice (*Smn*^ko/ko^; huSMN2^tg/0^) and 50% of control mice (*Smn*^ko/wt^; SMN2^tg/0^), on the FVB/NRj background (Janvier, Le Genest-St-Isle, France) following 10 generations of backcrossing. All animals were housed in temperature- and humidity-controlled rooms with ad libitum access to food and water. Daily injections from birth were administered to SMA and control heterozygous mice with either flunarizine (500 µg/mL, 1 µL/g) or vehicle (1% DMSO in saline solution) as described [28]. A unique identifier was assigned to each animal, and no selection was made among mice since the SMA phenotype was not yet visible at birth. Experiments were conducted in a blinded manner for genotype, treatment, and molecular studies unless otherwise specified. Group allocation was disclosed for analyses, and no exclusion criteria were applied. Both females and males were included in the study. Three to six mice per experimental group were studied based on previous results to minimize animal use [21]. Mice were genotyped as described [95]. At P10, SMA mutants and their heterozygote littermates were anesthetized by intraperitoneal injections of pentobarbital (64 mg/kg) and decapitated, and tissues were dissected, snap-frozen, and stored at −80 °C for RNA extractions. For immunofluorescence experiments, spinal cords were dissected at P5 and post-fixed for 16 h, embedded in agarose, and sectioned. Sections (50 μm) were incubated in PBS containing 0.01 M glycine for 1 h, washed in TBS-T (0.5% Tween), blocked with 0.5% Triton X-100, 4% FBS, and 1% BSA in TBS-T for 1 h, and followed by an incubation for 72 h at 4 °C with primary antibodies (Table S3) diluted in blocking solution. Sections were washed in TBS-T and incubated for 1 h with secondary antibodies diluted in TBS-T containing 0.5% Triton X-100, 0.4% FBS, and 0.1% BSA. After incubation with bisbenzimide, sections were washed and mounted with fluoromount-G (Invitrogen).

### 4.8. Immunofluorescence Microscopy, Image Acquisition, Processing, and Analysis

Samples were imaged with an ORCA Flash camera (Hamamatsu Photonic) mounted on an epifluorescence microscope (Axio Observer Z1, ZEISS) using either a 20× or 63× oil-immersion objective. ZEN software was used to acquire and analyze the images. Figures were prepared using either ImageJ or ZEN software.

### 4.9. Statistical Analysis

At least three independent experiments were performed and presented as the mean ± SD or SEM. Statistical analyses were conducted using Graphpad Prism 9.5.0. A normality test was conducted, and statistical tests were indicated in figure legends. *p*-values ≤ 0.05 were considered statistically significant.

## Data Availability

The datasets supporting the conclusions of this article are included within the article and its Supplemental Information.

## Supplementary Materials

The following supporting information can be downloaded at: https://www.mdpi.com/1422-0067/25/18/10039/s1.
