# Supplementary material for: Understanding the Role of the SMN Complex Component GEMIN5 and Its Functional Relationship with Demethylase KDM6B in the Flunarizine-Mediated Neuroprotection of Motor Neuron Disease Spinal Muscular Atrophy
Source: Int J Mol Sci. 2024 Sep 18;25(18):10039. doi: 10.3390/ijms251810039 (PMC11431868; doi:10.3390/ijms251810039)

SUPPLEMENTAL INFORMATION

UNDERSTANDING THE ROLE OF SMN COMPLEX COMPONENT GEMIN5 AND ITS FUNCTIONAL RELATIONSHIP WITH DEMETHYLASE KDM6B IN FLUNARIZINE-MEDIATED NEUROPROTECTION OF MOTOR NEURON DISEASE SPINAL MUSCULAR ATROPHY

Badih Salman[1], Emeline Bon[1], Perrine Delers[1], Steve Cottin[1], Elena Pasho[2], Sorana Ciura[2], Delphine Sapaly[1] and Suzie Lefebvre[1*]

[1]Université Paris Cité, T3S, INSERM UMR1124, Faculté des Sciences Fondamentales et Biomédicales. F – 75006, Paris, France.

[2]Université Paris Cité, INSERM UMR1163, Institut Imagine. F – 75015, Paris, France.

* Correspondence: suzie.lefebvre@inserm.fr

Supplemental Table 1. Gene changes (FC>1.3 ; p<0.05) in SMA fibroblasts treated with flunarizine[27]

ID	description	p-value	Fold Change
TXNIP	thioredoxin interacting protein	5,83E-08	-2,130946545
ARRDC4	arrestin domain containing 4	1,36E-04	-1,737334089
KRTAP2-3	keratin associated protein 2-3	1,50E-04	1,774785346
EGR1	early growth response 1	7,86E-04	1,625826817
HIST3H2A	histone cluster 3, H2a	0,001323784	-1,64062971
RPL21	ribosomal protein L21	0,001398914	-1,54876945
MT-ND1	mitochondrially encoded NADH dehydrogenase 1	0,001417832	1,612187443
MT-ND2	mitochondrially encoded NADH dehydrogenase 2	0,003097574	1,558065964
PTGS2	prostaglandin-endoperoxide synthase 2	0,003107159	1,504157578
MRPL12	mitochondrial ribosomal protein L12	0,003737092	-1,563047653
COX5A	cytochrome c oxidase subunit 5A	0,003883642	1,557634867
MT-ND6	mitochondrially encoded NADH dehydrogenase 6	0,004299263	1,523714306
ALDOAP2	ALDOA pseudogene 2	0,004340983	1,546780968
RPL3P12	ribosomal protein L3 pseudogene 12	0,004424532	-1,541059237
MT-ND5	mitochondrially encoded NADH dehydrogenase 5	0,005248828	1,52114922
SERPINB2	serpin family B member 2	0,006928108	1,454766838
YDJC	chitooligosaccharide deacetylase homolog	0,006957187	-1,483141589
CD14	CD14 molecule	0,007071921	-1,481897867
TERC	telomerase RNA component	0,00719568	-1,462339379
MT-ATP8	mitochondrially encoded ATP synthase 8	0,007588575	1,508173413
RPS29	ribosomal protein S29	0,008902873	-1,455610151
POTEF	POTE ankyrin domain family member F	0,010259466	1,485398893
IL6	interleukin 6	0,01045389	1,449203129
KDM6B	lysine demethylase 6B	0,010749106	1,463568039
RAB44	RAB44, member RAS oncogene family	0,011768913	-1,231752773
ADAMTSL4-AS1	ADAMTSL4 antisense RNA 1	0,012680608	1,395605774
STX3	syntaxin 3	0,012727788	1,437234292
C2orf61	STPG4 sperm-tail PG-rich repeat containing 4	0,012996675	-1,224944067
GDNF	glial cell derived neurotrophic factor	0,0145664	1,437003196
MXD3	MAX dimerization protein 3	0,014854036	-1,445442993
EIF5B	eukaryotic translation initiation factor 5B	0,014994956	1,422007144
SRRM2	serine/arginine repetitive matrix 2	0,016664918	1,411549648
IFI44L	interferon induced protein 44 like	0,018050207	1,429222229
MT2P1	metallothionein 2 pseudogene 1	0,018649871	1,428194046
JOSD2	Josephin domain containing 2	0,018841929	-1,43115978
FTH1P2	ferritin heavy chain 1 pseudogene 2	0,018945733	1,435384837
CAPN14	calpain 14	0,018972415	-1,225503281
TMEM40	transmembrane protein 40	0,019751446	-1,183200287

GEM	GTP binding protein overexpressed in skeletal muscle	0,020490288	1,413729005
LDHD	lactate dehydrogenase D	0,0216449	-1,33392841
MT-TW	mitochondrially encoded tRNA tryptophan	0,022844512	-1,418933014
TMEM155	SMIM43 small integral membrane protein 43	0,023003543	-1,41387463
NFATC2	nuclear factor of activated T cells 2	0,023052178	1,414605418
DEC1	BHLHE40 basic helix-loop-helix family member e40	0,023214117	-1,263059742
MAP1LC3B2	microtubule associated protein 1 light chain 3 beta 2	0,023705014	1,408536763
FOSL1	FOS like 1, AP-1 transcription factor subunit	0,025004283	1,396721681
HNRNPA1P5	heterogeneous nuclear ribonucleoprotein A1 pseudogene 5	0,025163104	-1,271971819
HIPK2	homeodomain interacting protein kinase 2	0,025193631	1,371176817
HIC1	HIC ZBTB transcriptional repressor 1	0,02535751	-1,383928695
ALDOAP1	ALDOA pseudogene 1	0,025702596	1,409778721
GBP4	guanylate binding protein 4	0,025790187	-1,24655695
CXCL2	C-X-C motif chemokine ligand 2	0,025825124	1,408606714
CYP1A1	cytochrome P450, family 1, subfamily a, polypeptide 1	0,025937736	1,408529832
DUSP6	dual specificity phosphatase 6	0,026130325	1,36213709
FAM173A	ANTKMT adenine nucleotide translocase lysine methyltransferase	0,026283653	-1,405598015
MT-TK	mitochondrially encoded tRNA lysine	0,026803123	-1,393119167
MEGF6	multiple EGF like domains 6	0,026842748	-1,369843916
ITGA11	integrin subunit alpha 11	0,027531587	1,382008639
FLG-AS1	FLG antisense RNA 1	0,027789856	-1,167081768
GJD2	gap junction protein delta 2	0,027789856	-1,167081768
XDH	xanthine dehydrogenase	0,028019403	1,34508407
TUSC1	tumor suppressor candidate 1	0,028142951	-1,385473298
MTND1P23	MT-ND1 pseudogene 23	0,028433063	1,402018665
CCDC85A	coiled-coil domain containing 85A	0,029274921	-1,39213022
RHBDL1	rhomboid like 1	0,029448329	-1,276814313
IER3	immediate early response 3	0,030014814	1,357228017
SHROOM2	shroom family member 2	0,03021994	-1,184241575
RGAG4	RTL5 retrotransposon Gag like 5	0,030641627	1,370971699
PVRL3-AS1	NECTIN3-AS1 NECTIN3 antisense RNA 1	0,030829455	-1,287576772
RPL35P1	ribosomal protein L35 pseudogene 1	0,030933138	-1,386943928
SEC63P1		0,031549458	1,390333451
NSUN2	NOP2/Sun RNA methyltransferase 2	0,031741578	1,36976065
ATP5D	ATP synthase F1 subunit delta	0,031825817	-1,364145982
CEACAM1	CEA cell adhesion molecule 1	0,031870828	-1,304523868
IER5L	immediate early response 5 like	0,032065655	-1,360632877
GPR68	G protein-coupled receptor 68	0,032086052	1,372129736
MT2A	metallothionein 2A	0,032143633	1,365186706
RRAGC	Ras related GTP binding C	0,03251342	1,368885543

ELN	elastin	0,032695823	-1,339826744
ALKBH7	alkB homolog 7	0,032921168	-1,359628719
DRC1	dynein regulatory complex subunit 1	0,033142022	-1,194432291
ID1	inhibitor of DNA binding 1	0,033586863	-1,34090691
LIF	LIF interleukin 6 family cytokine	0,033787863	1,376233948
GDF15	growth differentiation factor 15	0,033848347	1,380749612
NRROS	negative regulator of reactive oxygen species	0,033874017	1,340518372
THBD	thrombomodulin	0,03457403	1,351248143
ACTG1P1	actin gamma 1 pseudogene 1	0,035674565	1,367634725
UQCRQ	ubiquinol-cytochrome c reductase complex III subunit VII	0,036452828	-1,344327501
CXCL3	C-X-C motif chemokine ligand 3	0,03766698	1,354150807
TP53TG1	TP53 target 1	0,037854783	-1,376397659
SIRPAP1	signal regulatory protein alpha pseudogene 1	0,038261289	1,34425669
ZNF787	zinc finger protein 787	0,038603423	-1,342417839
LRP3	LDL receptor related protein 3	0,039358838	-1,330000959
FAM47ESTBD1	FAM47E-STBD1 readthrough	0,039468148	-1,372822358
PDXP	pyridoxal phosphatase	0,040582527	-1,339937615
TMEM261	DMAC1 distal membrane arm assembly component 1	0,040702842	-1,338521748
MASP2	MBL associated serine protease 2	0,041477805	-1,158631045
FAM103A1	RAMAC RNA guanine-7 methyltransferase activating subunit	0,041589485	1,368129591
ZFP36L1	ZFP36 ring finger protein like 1	0,041949357	1,33043421
ADAMTS5	ADAM metallopeptidase with thrombospondin type 1 motif 5	0,042233775	-1,328706388
SLC35G5	solute carrier family 35 member G5	0,04245381	-1,194414092
C19orf70	MICOS13 mitochondrial contact site and cristae organizing system subunit 13	0,042764117	-1,335045523
GREM1	gremlin 1, DAN family BMP antagonist	0,043443653	1,33569002
CBX8	chromobox 8	0,04397571	-1,364237618
SIVA1	SIVA1 apoptosis inducing factor	0,044241882	-1,321265138
BCORL1	BCL6 corepressor like 1	0,044458841	1,348279464
AP3B2	adaptor related protein complex 3 subunit beta 2	0,045192626	-1,350413725
CWC27	CWC27 spliceosome associated cyclophilin	0,045543999	1,355844782
MRPS12	mitochondrial ribosomal protein S12	0,045549953	-1,327137156
POU2F2	POU class 2 homeobox 2	0,046213236	1,346576719
PRKXP1	protein kinase cAMP-dependent X-linked catalytic subunit (PRKX) pseudogene 1	0,046529137	1,313312107
HDHD3	haloacid dehalogenase like hydrolase domain containing 3	0,046719592	-1,350940989
ARHGEF33	Rho guanine nucleotide exchange factor 33	0,046949442	1,298999007
HSPA8P8	heat shock protein family A (Hsp70) member 8 pseudogene 8	0,04742053	1,329516268
ENDOG	endonuclease G	0,047491571	-1,33118551
EEF1A1P4	eukaryotic translation elongation factor 1 alpha 1 pseudogene 4	0,047912391	-1,353310306

NDUFB7	NADH:ubiquinone oxidoreductase subunit B7	0,048387721	-1,322287774
EID2	EP300 interacting inhibitor of differentiation 2	0,048971542	-1,326171857
MLXIP	Max-like protein X (MLX) interacting protein	0,049324646	1,332484782

Supplemental Table 2. Primers used for qPCR analysis.

	Sequence (5'-3')	Efficiency	R^2	Source
Smn E4-5-F<br>Smn E4-5-R	5'-TGGATATGGAAACAGAGAGGAG-3'<br>5'-GGTGGTGGAGGAAGAAATGAG-3'	96%	0,997	herein
Gemin3-F<br>Gemin3-R	5'-GCTCTGGACTCTCTGATTCTTG-3'<br>5'-ATGGCTGTGATAACCGAGTG-3'	99%	0,9958	herein
Gemin5 E4-6F<br>Gemin5 E4-6-R	5'-AGACTGCTTATCCATCAGCC-3'<br>5'-ACCCCCACTTCTTCTCTTC-3'	95%	0,9942	herein
Gemin6-F<br>Gemin6-R	5'-AGTCTCTGCCAACATTGTCC-3'<br>5'-TATGGTTTCCACAGTCTGCAC-3'	84%	0,9923	herein
Strap E1-2-F<br>Strap E1-2-R	5'-GTGGTGGATTTGGCCTTCAG-3'<br>5'-GGCATCCTTATTCAATGTTGCAC-3'	88%	0,9986	PrimerBank<br>ID 6755682a1
Strap E4-6-F<br>Strap E4-6-R	5'-ACTGGGGGACAGGATAAAC-3'<br>5'-CTCCATGCTGCTAACAGAC-3'	99%	0,9584	herein
Strap E8-9-F<br>Strap E8-9-R	5'-CCATCAATTCTGCGTCTCTTC-3'<br>5'-GCCACAATCTCAATGTCCC-3'	104%	0,9973	herein
Strap E4-5-F<br>Strap E4-5-R	5'-CCTGAAGCAGAACCTAAGGAAA-3'<br>5'-ATCATCCGCTGAAAGGATCTG-3'	101%	0,9988	herein
Strap E5-6-F<br>Strap E5-6-R	5'-CTGTGGTGCAGTGACGATAA-3'<br>5'-TCCATGCTGCTAACAGACATATT-3'	97%	0,9949	herein
Kdm6b E1-2-F<br>Kdm6b E1-2-R	5'-ACCCCACTTCTGCTGTAACC-3'<br>5'-GCCAATCATCACCCTTGTCTC-3'	108%	0,9928	herein
Kdm6b E11-12-F<br>Kdm6b E11-12-R	5'-AAGGTGGAAAGTGGGGACAAGG-3'<br>5'-TTGGGTTTCACAGACTGGGC-3'	88%	0,9995	herein
Kdm6b E19-20-F<br>Kdm6bE19-20-R	5'-TGAAGAACGTCAAGTCCATTGTG-3'<br>5'-TCCCGCTGTACCTGACAGT-3'	101%	0,9946	PrimerBank<br>ID 20809817a1
Fnip1E9-10-F<br>Fnip1E9-10-R	5'-AGGAAGAGAACTGAATGAGGAC-3'<br>5'-GAAACACCCCATTTTCCAAAC-3'	104%	0.9926	herein
Fnip1E16-17-F<br>Fnip1E16-17-R	5'-TGGACAGAAGAGGATGAGATAC-3'<br>5'-GTCAGGAACATAAGATGAGCAG-3'	94%	0.9991	herein
Fus-F<br>Fus-R	5'-GCAGCACCTCAGGAAGTTAT-3'<br>5'-CACCATAGCCAGACTGTTGA-3'	100%	0,997	herein
TDP43-F<br>TDP43-R	5'-CCAATGCTGAACCTAAGCATAATAG-3'<br>5'-GGATGAGAAAGCATGTAGACAGA-3'	96%	0,9953	herein
Txnip-F<br>Txnip-R	5'-GTGTCCCTGGCTCCAAGAAA-3'<br>5'-GAGAGTCGTCCACATCGTCC-3'	97%	0.9979	herein

Egr1-F<br>Egr1-R	5’-AATAGCAGCAGCAGCACCAG-3’<br>5’-ATAACTCGTCTCCACCATCGCC-3’	96%	0,9965	herein
Dusp6-F<br>Dusp6-R	5’-ATAGATACGCTCAGACCCGTG-3’<br>5’-ATCAGCAGAAGCCGTTCGTT-3’	84%	0,9912	PrimerBank<br>ID 13399314a1
Lif E4-5-F<br>Lif E4-5-R	5’-CAGATCAAGAATCAACTGGCAC-3’<br>5’-ACCATCCGATACAGCTCCAC-3’	95%	0,9801	herein
Stx3-F<br>Stx3-R	5’-TCGGATACGAAAGTCCCAG-3’<br>5’-CCACTCTCCAACATCTCTTCC-3’	97%	0,9873	herein
Hipk2-F<br>Hipk2-R	5’-TGAACCAGAGCAAAACACC-3’<br>5’-AGTATGGAGACTTCGGGATTG-3’	102%	0,999	herein
Agrin-F<br>Agrin-R	5’-GCAAGAAGAATGTCTGCCC-3’<br>5’-AGTCAACACCATCACTGCC-3’	81%	0.9964	herein
Nme1-F<br>Nme1-R	5’-AGGAGCACTACACTGACCTGA-3’<br>5’-GGTTGGTCTCTCCAAGCATCA-3’	90%	0,9984	PrimerBank<br>ID 1816594a1
Siva1 E1-2-F<br>Siva1 E1-2-R	5’-CCGCTCCAACTCAAAGTCCA-3’<br>5’-CGGAACAATCTTCGCTCGATATG-3’	97%	0,9895	PrimerBank<br>ID 7305493a1
Mnx1-F<br>Mnx1-R	5’-CACCAGTTCAAGCTCAACAAG-3’<br>5’-CATTTCATTCGGCGGTTCTG-3’	104%	0,9972	herein
Isl1-F<br>Isl1-R	5’-GGCGATCCACCAAAAAAAAAAC-3’<br>5’-CCCATCCCTAACAAAGCAC-3’	100%	0,993	herein
Lhx3-F<br>Lhx3-R	5’-GCAGGTGTGGTTCCAGAAT-3’<br>5’-GCGGGAGCGCTTCATATT-3’	88%	0,9994	herein
Foxp1-F<br>Foxp1-R	5’-TTTAATCAGGCAGGCCATTC-3’<br>5’-GCATTGCGTCGGAAGTAAG-3’	113%	0,9935	herein
Il6-F<br>Il6-R	5’TCACTTTGAGATCTACTCGGCAAACC3’<br>5’-TCTGACCACAGTGAGGAATGTCCA-3’	81%	0,9921	herein
Gdnf-F<br>Gdnf-R	5’-CTGACTTGGGTTTGGGCTATGA-3’<br>5’-TGCCTGGCCTACTTTGTCACTT-3’	128%	0.9962	Zhao et al. 2017. J of Neurosci 37 (13) 3465-3477.
Gdf15-F<br>Gdf15-R	5’-GAGCTACGGGGTCGCTTC-3’<br>5’-GGGACCCCAATCTCACCT-3’	104,5%	0,9973	Plomgaard et al. 2022. Front Endocrinology 13 (Dec) : 1037948
Cbx8-F<br>Cbx8-R	5’-ATTCGCAAAGGACGCATGGAA-3’<br>5’-CCTCGCTTTTTGGGGCCATA-3’	86%	0,9958	PrimerBank<br>ID 7304947a1
Bcorl1-F<br>Bcrol1-R	5’-AGTCAGGGGAAAGCACAAG-3’<br>5’-TCAGAACCACAGGGATGAAG-3’	112%	0.9987	herein
Cwc27-F<br>Cwc27-R	5’-AGCATGAAGGGGAGAAGTAAG-3’<br>5’-GCACTGTCATCCTCACCATC-3’	98%	0,9989	herein
Rpl13a-F<br>Rpl13a-R	5’-AGGGGCAGGTTCTGGTATTG-3’<br>5’-TGTTGATGCCTTCACAGCGT-3’	89%	0,997	Gong et al. 2016. Sc Reports 6
Sdha-F<br>Sdha-R	5’-GGAACACTCCAAAAACAGACCT-3’<br>5’-CCACCACTGGGTATTGAGTAGAA-3’	92%	0,9972	Van Deventer et al.2008. Am J Pathol 173 (1): 253–64.
Ppia-F<br>Ppia-R	5’-GGCAAATGCTGGACCAAAC-3’<br>5’-CATTCCTGGACCCAAAACG-3’	102%	0,9996	Gong et al. 2016. Sc Reports 6
Actb-F<br>Actb-R	5’-CCTTCTTGGGTATGGAATCCTGT-3’<br>5’CACTGTGTTGGCATAGAGGTCTTTAC3’	90%	0,9987	Gong et al. 2016. Sc Reports 6
Hmbs-F<br>Hmbs-R	5’-ATGAGGGTGATTCGAGTGGG-3’<br>5’-TTGTCTCCCGTGGTGGACATA-3’	87%	0,9975	Gong et al. 2016.

Hprt1-F<br>Hprt1-R	5’-TCAGTCAACGGGGGACATAAA-3’<br>5’-GGGGCTGTACTGCTTAACCAG-3’	90%	0,998	Sommer et al. 2016. *Journal of Neuroinflammation* 13(1): 174.
Gapdh-F<br>Gapdh-R	5’-AAGGGCTCATGACCACAGTC-3’<br>5’-ACACATTGGGGGTAGGAACA-3’	71%	0,9999	Li, Xu, and Pozzo-Miller. 2016. PNAS 113(11): E1575–84.

Supplemental Table 3. Antibodies used for immunodetection

Target	Source	RRID	Host species	Concentration used	
				IHC	immunoblot
SMN	502 (homemade)	NA	rabbit		1:500
SMN	610647 (BD Transduction Laboratories)	AB_397973	mouse	1:400	
GEMIN2	Sc-33703 (Sta Cruz)	AB_627668	mouse		1:400
GEMIN3	GTX54029(GeneTex)	GTX54029	rabbit		1:500
GEMIN5	GTX130498(GeneTex)	AB_2886289	rabbit		1:500
GEMIN8	Sc-68374(Santa-Cruz)	AB_2111849	rabbit		1:500
UNRIP	Ab205015(Abcam)	NA	rabbit		1:2000
DUSP6	SAB5700869(Merck)	NA	rabbit		1:1000
KDM6B	ab169197 (Abcam)	NA	rabbit	1:100	1:500
FNIP1	28380-1-AP(proteintech)	AB_2881128	rabbit		1:500
HB9	Sc-515769 (Sta Cruz)	NA	mouse	1:50	1:100
Mouse Fc Block	Ms CD16/CD32 2.4G2	AB_394656	rat	1:50	
ChAT	AB144P(Sigma-Aldrich)	AB_2079751	Goat	1:100	
TDP-43	10782-2-AP(proteintech)	AB_615042	rabbit		1:10000
FUS	11570-1-AP(proteintech)	AB_2247082	rabbit		1:1000
HIPK2	PA5-40567(Invitrogen)	AB_2605444	rabbit		1:500
Coilin	SC-32860 (Sta Cruz)	AB_2081431	Rabbit	1:500	
Tubulin alpha	B-5-1-2 (Invitrogen)	NA	mouse		1:5000
Clean blot-IP	21230 (ThermoFisher Scientific)	NA			1:2000-1:4000
Anti-mouse antibody(Cy3)	112-166-062 (Jackson ImmunoResearch)	AB_2338254	goat	1:400	
Anti-rabbit antibody (AF488)	A11034(ThermoFisher Scientific)	AB_2576217	goat	1:400	
Anti-goat antibody (AF546)	A-11056 (LifeTechnologies)	AB_2534103	donkey	1:400	
Anti-mouse antibody (AF488)	715-545-150 (Jackson ImmunoResearch)	AB_2340846	donkey	1:200	
Anti-rabbit antibody (AF647)	711-605-152 (Jackson ImmunoResearch)	AB_2492288	donkey	1:400	

SUPPLEMENTAL FIGURE LEGENDS

Figure S1. Differential gene expression of RNA targets in flunarizine-treated NSC34 cells RT-qPCR analysis of SMA fibroblasts candidate genes in NSC34 cells following different incubation times with flunarizine compared to corresponding DMSO treatment (arbitrary value of 1). Two internal control genes were used for normalization (30min-1h-2h: Rpl13a, Hmbs; 5h-16h: Sdha, Hprt1). Errors bars indicate the SD ($3 \leq n \leq 8$ independent experiments, one-way Anova followed by Dunnet's multiple t-test, « ns » not significant (P > 0.05, *P < 0.05, ** < 0.01).

Figure S2. Similar splicing modulation of *Kdm6b* and *Strap* transcripts by *Gemin5* depletion and 1-hr flunarizine treatment
(A) RT-qPCR analysis of Kdm6b exons 1-2 and exons 11-12 expression in NSC34 cells treated with flunarizine for the indicated time points compared to DMSO treatment (arbitrary value of 1). Two internal control genes were used for normalization (1h-2h: Rpl13a, Hmbs; 5h-16h: Sdha, Hprt1). Errors bars indicate the SD ($3 \leq n \leq 8$ independent experiments, one-way Anova followed by Dunnet's multiple t-test, « ns » not significant (P > 0.05, *P < 0.05, ** < 0.01, *** P < 0.001, **** < 0.0001). **(B)** RT-qPCR analysis of Kdm6b exons 1-2, exons 11-12, and exons 19-20 expression in NSC34 cells with the indicated treatments. DMSO and Si Neg were given an arbitrary value of 1. Two internal control genes were used for normalization (1h: Rpl13a, Hmbs; si*Gemin5*: Actb, Hmbs). Errors bars indicate the SD ($3 \leq n \leq 8$ independent experiments, one sample t-test, « ns » not significant (P > 0.05, *P < 0.05, ** < 0.01, *** P < 0.001). **(C)** Schematic representation of Kdm6b gene organization, isoforms and placement of RT-qPCR primers. **(D)** RT-qPCR analysis of Strap exons 1-2, exons 4-6, and exons 7-8 expression in NSC34 cells treated with flunarizine for the indicated time points compared to DMSO treatment (arbitrary value of 1). Errors bars indicate the SD ($3 \leq n \leq 8$ independent experiments, one-way Anova followed by Dunnet's multiple t-test, « ns » not significant (P > 0.05, *P < 0.05, **** < 0.0001). **(E)** RT-qPCR analysis of Strap exons 1-2, exons 4-6, exons 4-5, exons 5-6 and exons 7-8 expression with the indicated treatments. Two internal control genes were used for normalization (1h: Rpl13a, Hmbs; si*Gemin5*: Actb, Hmbs). DMSO and Si Neg were given an arbitrary value of 1. Errors bars indicate the S.D ($3 \leq n \leq 8$ independent experiments, one sample t-test, « ns » not significant (P > 0.05, *P < 0.05, ** < 0.01, *** P < 0.001, **** < 0.0001). **(F)** Schematic representation of Strap gene organization and placement of RT-qPCR primers. **(G)** RT-qPCR analysis of Fnip1 exons 9-10 and exons 16-17 expression in NSC34 cells treated with flunarizine for the indicated time points compared to DMSO treatment (arbitrary value of 1). Two internal control genes were used for normalization (1h2h: Rpl13a, Hmbs; 5h-16h: Sdha, Hprt1). Errors bars indicate the SD ($3 \leq n \leq 8$ independent experiments, one-way Anova followed by Dunnet's multiple t-test, « ns » not significant (P > 0.05, *P < 0.05, ** < 0.01, *** P < 0.001, **** < 0.0001). **(H)** RT-qPCR analysis of Fnip1 exons 9-10, exons 16-17 expression in NSC34 cells treated with the indicated treatments. Two internal control genes were used for normalization (1h: Rpl13a, Hmbs; s*iGemin5*: Actb, Hmbs). DMSO and Si Neg were given an arbitrary value of 1. Errors bars indicate the SD ($3 \leq n \leq 8$ independent experiments, one sample t-test, « ns » not significant (P > 0.05, *P < 0.05, ** < 0.01, *** P < 0.001, **** < 0.0001). **(I)** Schematic representation of Fnip1 gene organization, isoforms and placement of RT-qPCR primers.

Figure S3. Key neurodevelopmental genes and SMN-complex components are modulated in the brain of *Smn*-deficient mice by flunarizine
(A) RT-qPCR analysis of candidate RNA targets in brain of vehicle (V)- or flunarizine (Flz)treated heterozygote control (CT) and SMN-deficient mice (SMA) at post-natal day P10. Internal control genes were used for normalization (Rpl13a, Actb, Ppia). Control vehicle was given an arbitrary value of 1. Error bars represented the standard deviation (SD) from the

mean values of triplicates from 3 ≤ n ≤ 6 mice per group. (One-way Anova followed by Turkey's multiple comparisons test. « ns » not significant (P > 0.05, *P < 0.05, ** < 0.01, *** P < 0.001.) **(B)** RT-qPCR analysis of genes encoding SMN-complex components in brain of vehicle (V)- or flunarizine (Flz)-treated heterozygote control (CT) and SMN-deficient mice (SMA) at postnatal day P10.

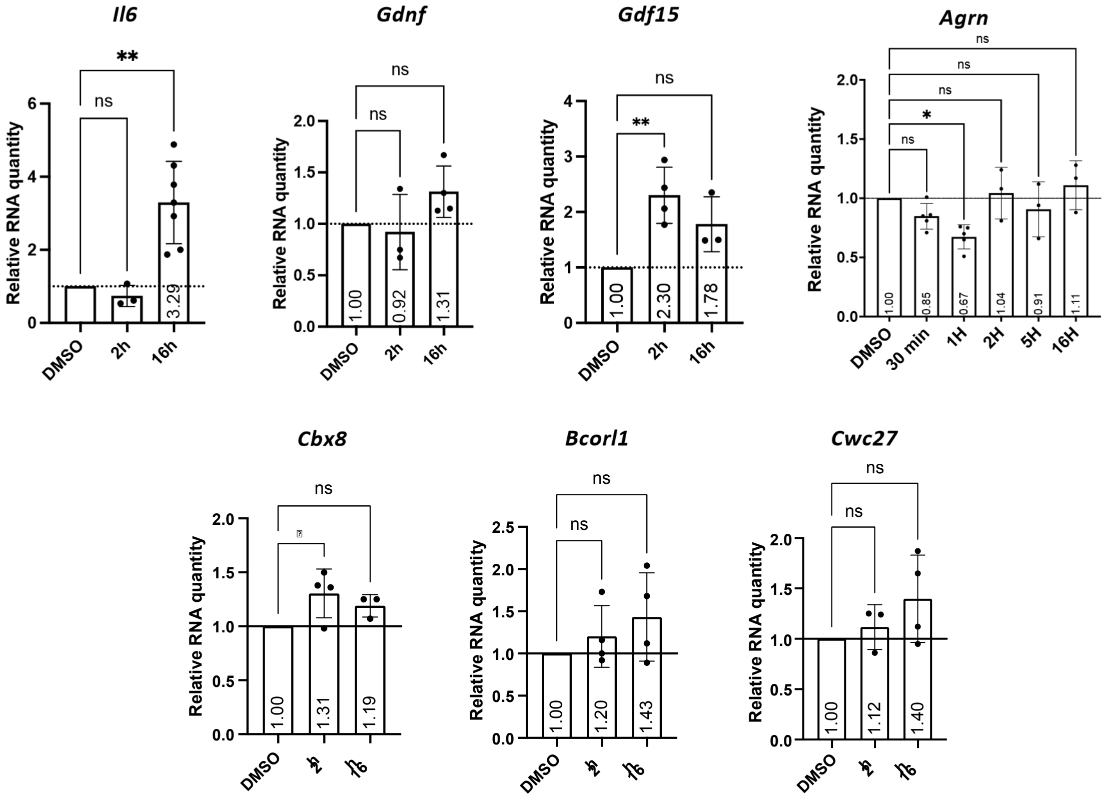

Il6
Gdnf
Gdf15
Agrn
Cbx8
Bcorl1
Cwc27
Relative RNA quantity
ns
**
*
DMSO
2h
16h
30 min
1H
2H
5H
16H
1.00
3.29
0.92
1.31
2.30
1.78
0.85
0.67
1.04
0.91
1.11
1.19
1.20
1.43
1.12
1.40

S1

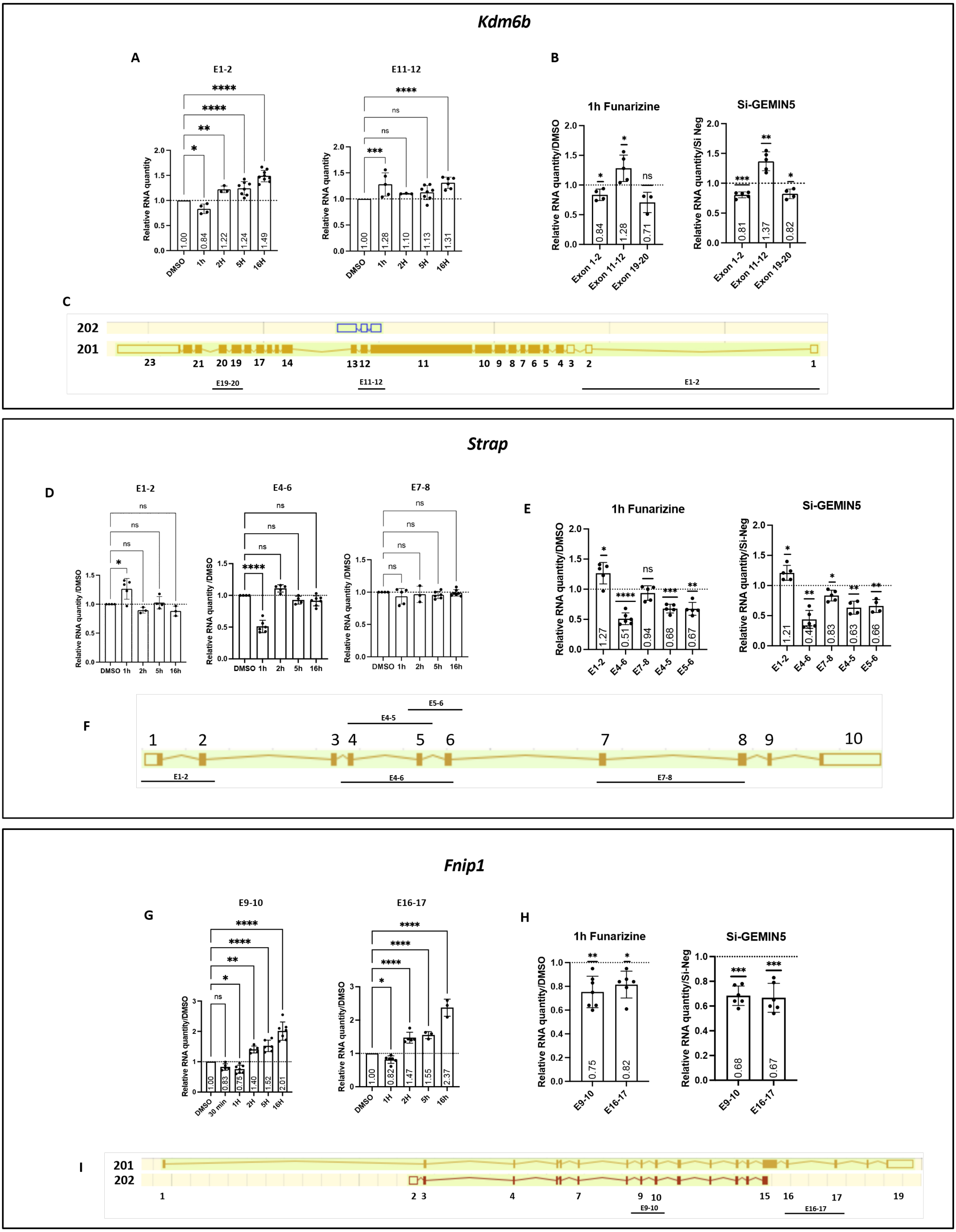
Kdm6b
A
E1-2
E11-12
Relative RNA quantity
DMSO
1h
2H
5H
16H
1.00
0.84
1.22
1.24
1.49
1.28
1.10
1.13
1.31
B
1h Funarizine
Si-GEMIN5
Relative RNA quantity/DMSO
Relative RNA quantity/Si Neg
Exon 1-2
Exon 11-12
Exon 19-20
0.84
1.28
0.71
0.81
1.37
0.82
C
202
201
E19-20
E11-12
E1-2
Strap
D
E1-2
E4-6
E7-8
Relative RNA quantity /DMSO
DMSO 1h 2h 5h 16h
E
1h Funarizine
Si-GEMIN5
Relative RNA quantity/DMSO
Relative RNA quantity/Si-Neg
E1-2
E4-6
E7-8
E4-5
E5-6
1.27
0.51
0.94
0.68
0.67
1.21
0.83
0.63
0.66
F
E5-6
E4-5
E1-2
E4-6
E7-8
Fnip1
G
E9-10
E16-17
Relative RNA quantity/DMSO
DMSO
30 min
1H
2H
5H
16H
1.00
0.83
0.75
1.40
1.52
2.01
0.82
1.47
1.55
2.37
H
1h Funarizine
Si-GEMIN5
Relative RNA quantity/DMSO
Relative RNA quantity/Si-Neg
E9-10
E16-17
0.75
0.82
0.68
0.67
I
201
202
E9-10
E16-17

S2

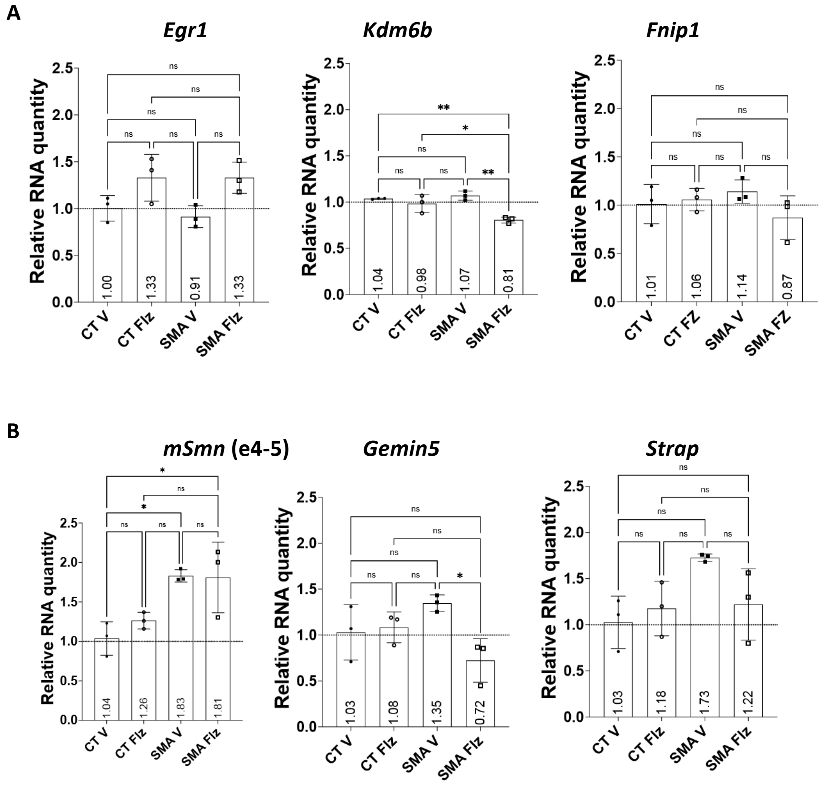
A
Egr1
Kdm6b
Fnip1
Dusp6
Lif
Relative RNA quantity
CT V
CT Flz
SMA V
SMA Flz
CT FZ
SMA FZ
1.00
1.33
0.91
1.33
1.04
0.98
1.07
0.81
1.01
1.06
1.14
0.87
1.00
1.08
1.10
1.24
1.09
0.98
1.26
2.15
ns
*
**
B
mSmn (e4-5)
Gemin5
Strap
1.04
1.26
1.83
1.81
1.03
1.08
1.35
0.72
1.03
1.18
1.73
1.22

S3

Following pages are the uncropped western blots and ponceau S red staining

In figure 1C, SMN

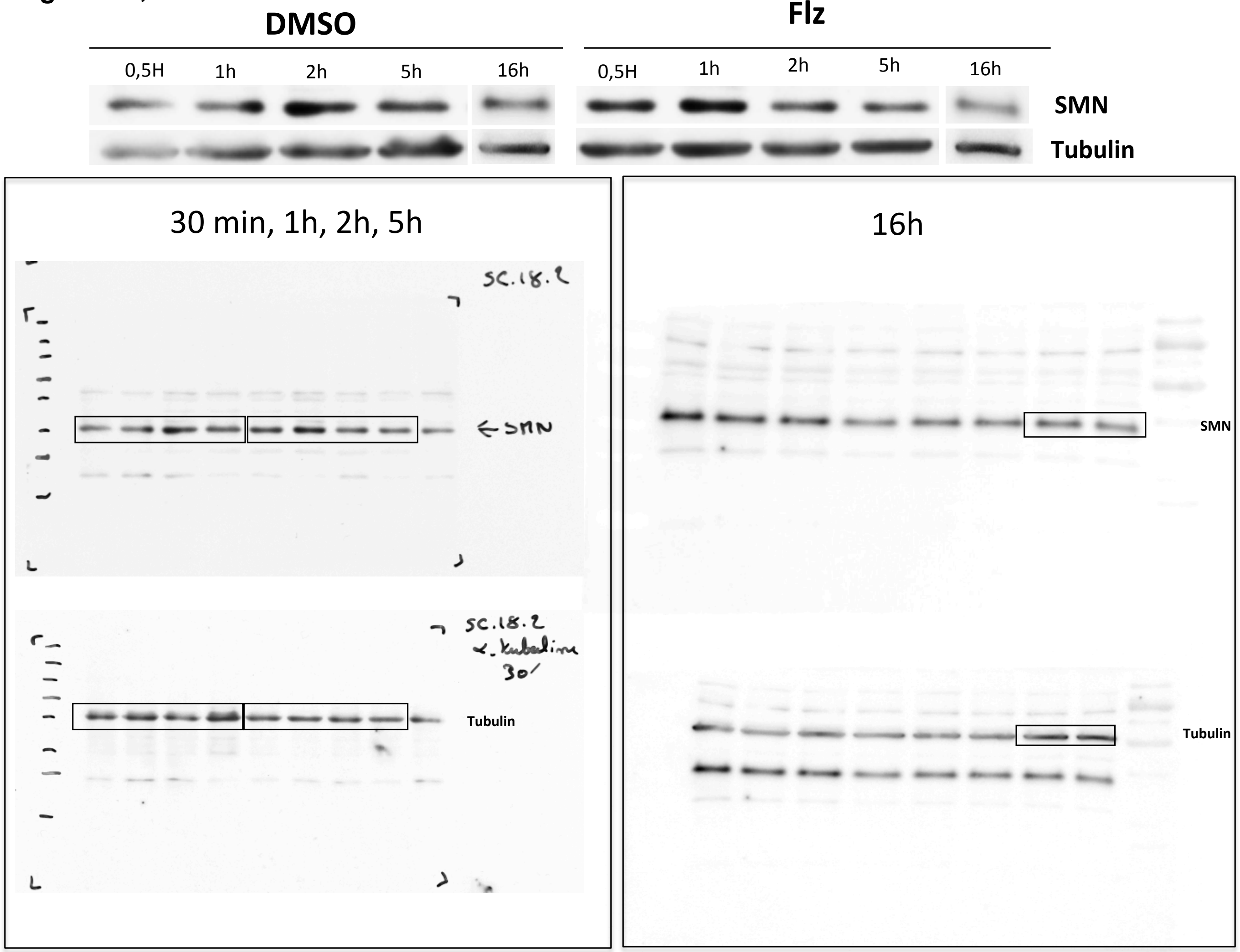

In figure 1C, Gemin3

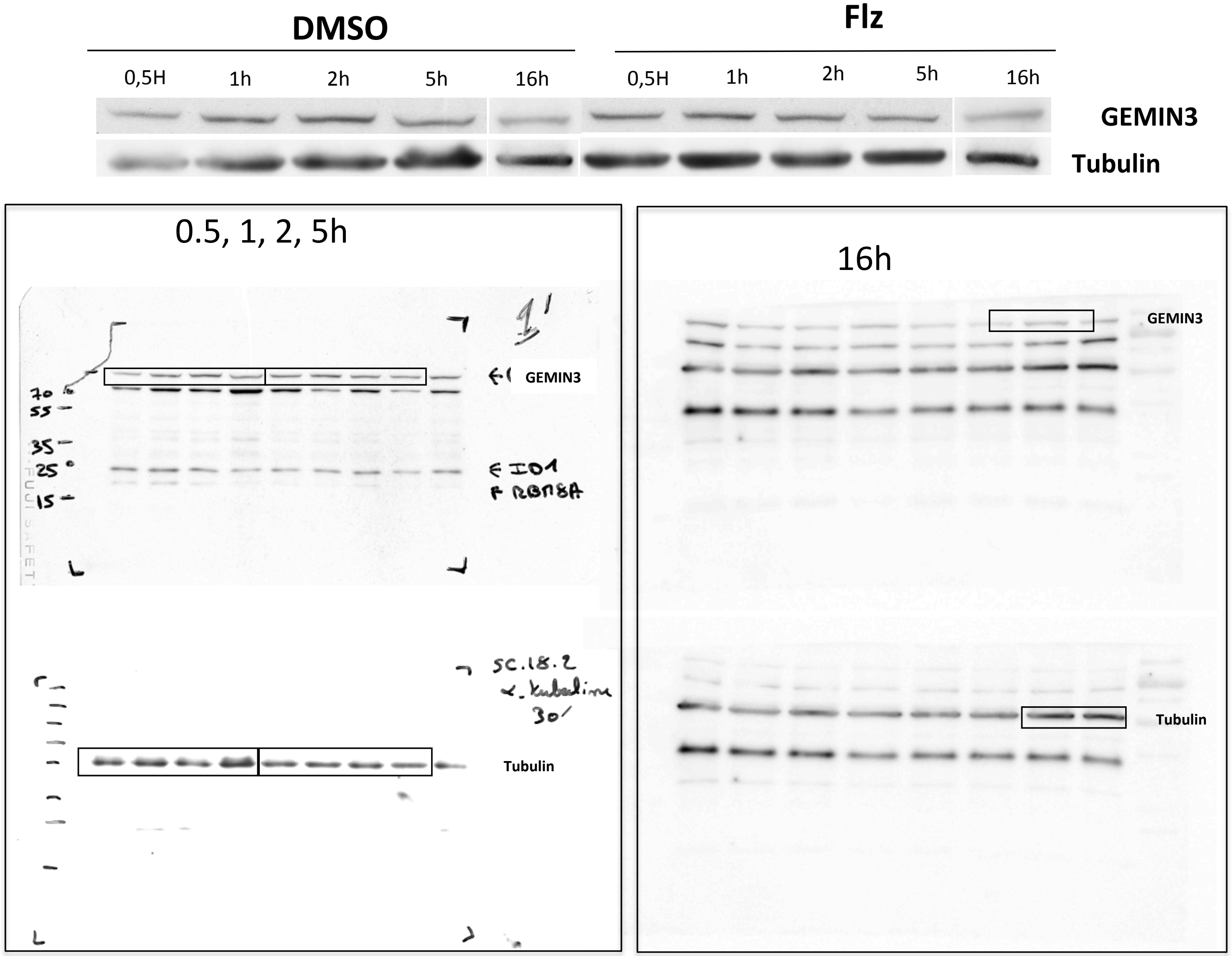

In figure 1C, GEMIN5

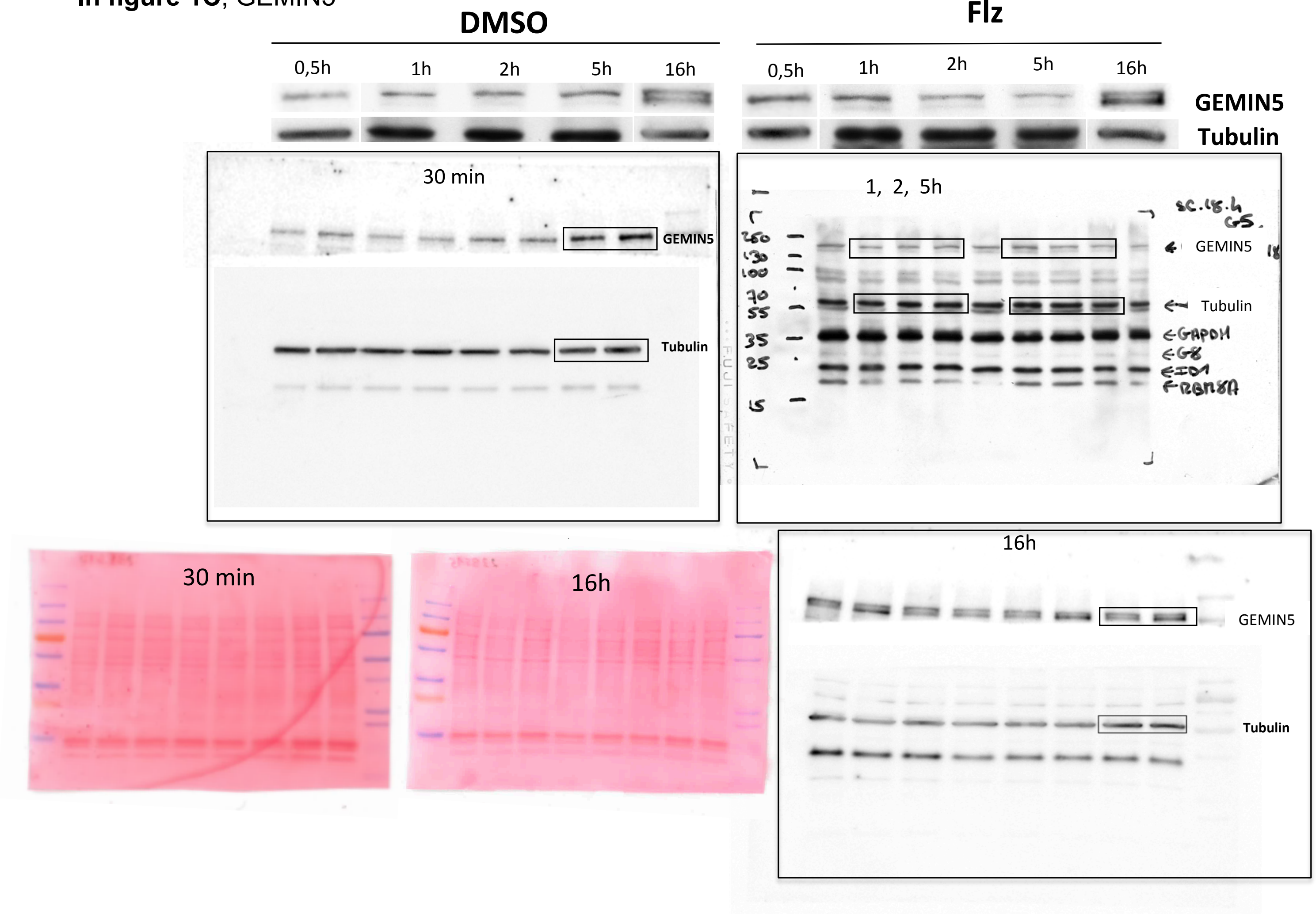

In figure 1C, STRAP

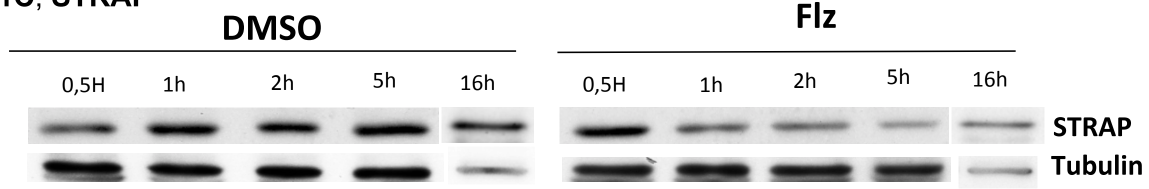

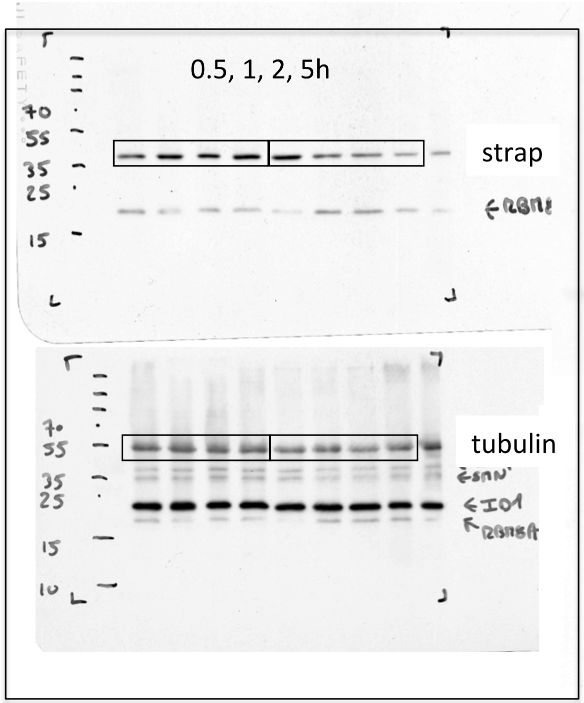

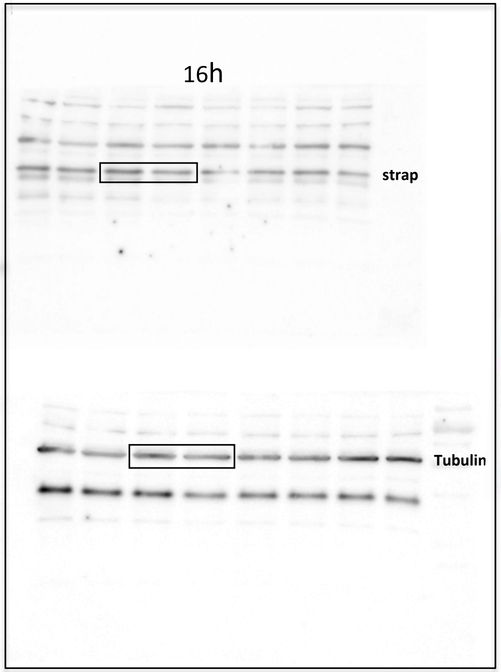

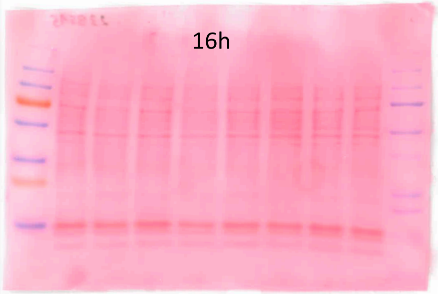

Figure 1G, DUSP6 – 30 min & 1 h

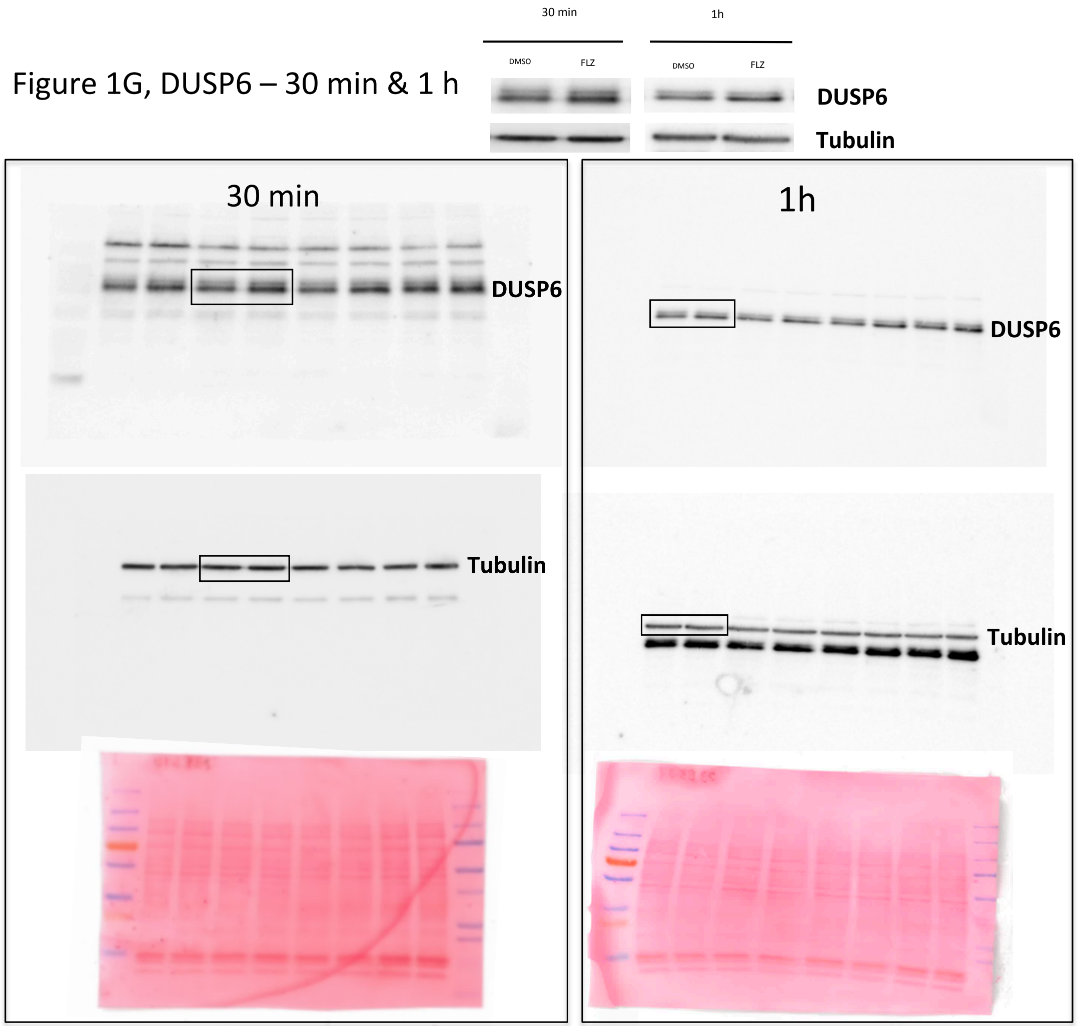

Figure 1G, DUSP6 – 2, 5 & 16h

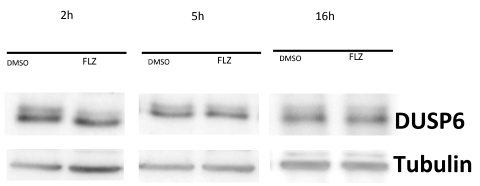

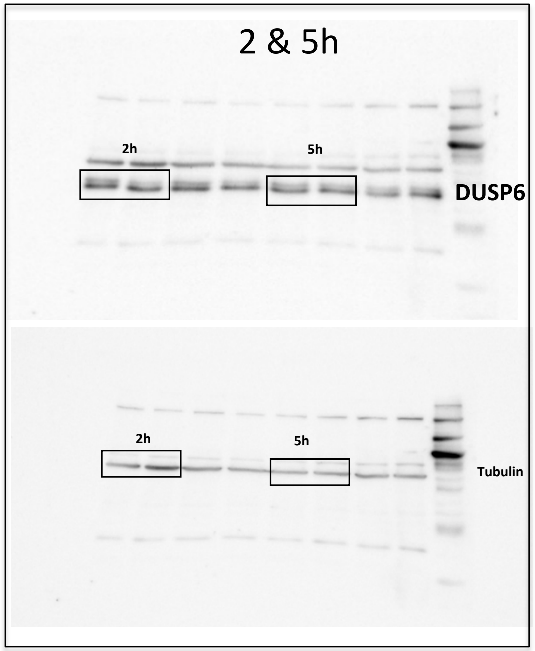

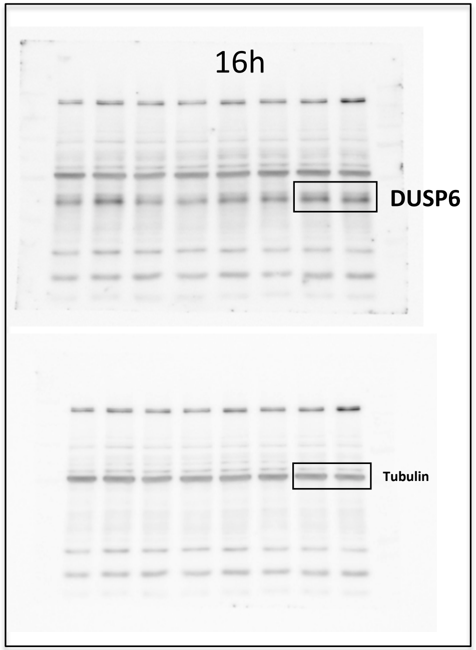

In figure 1G, KDM6B

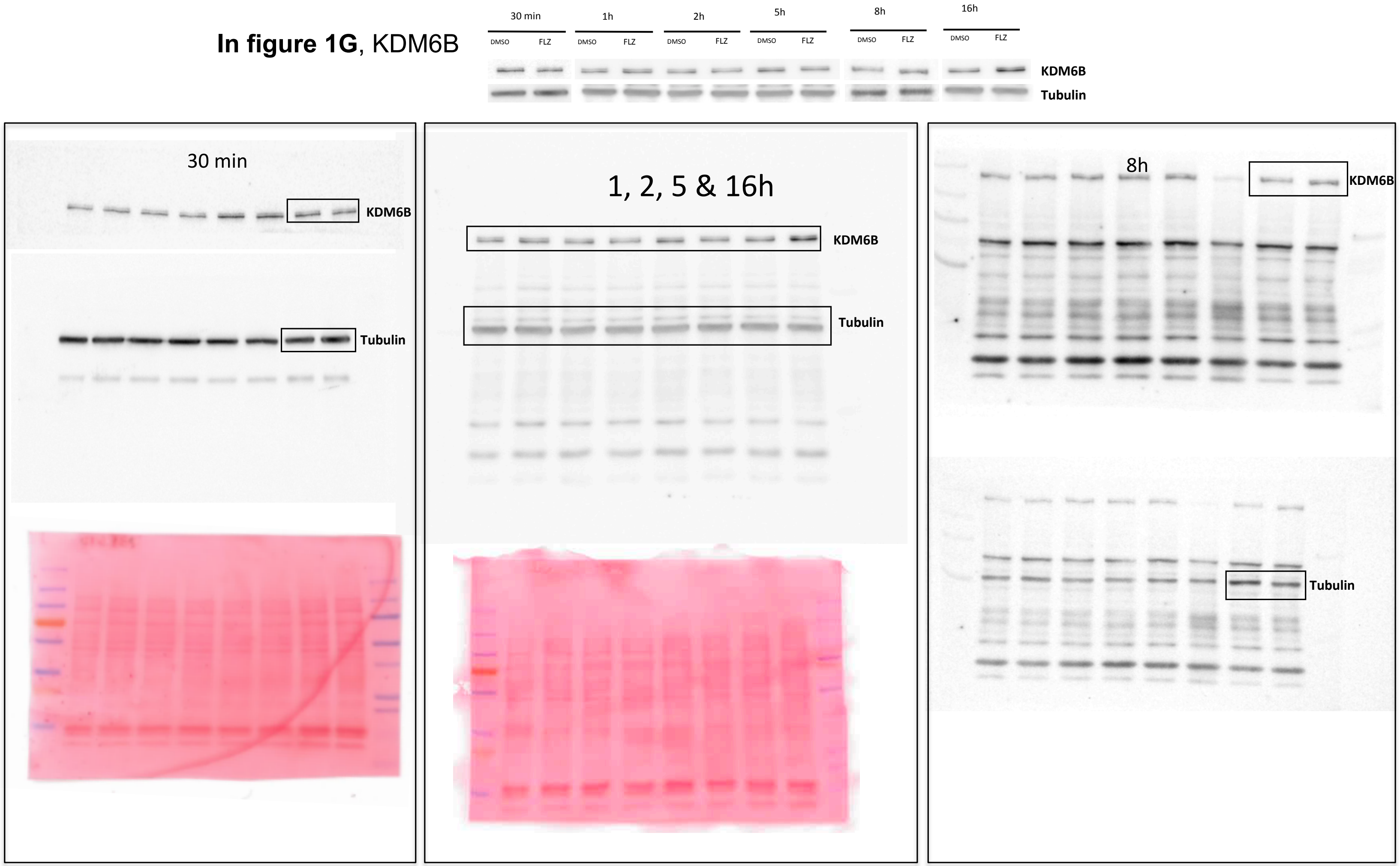

in figure 1G, HIPK2

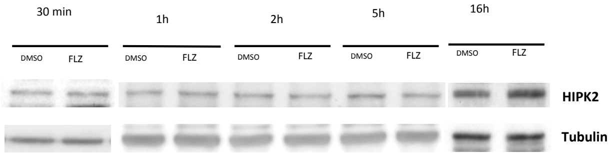

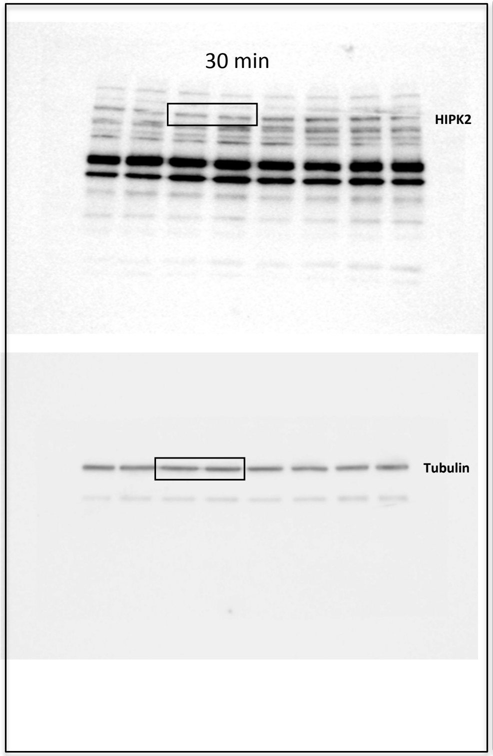

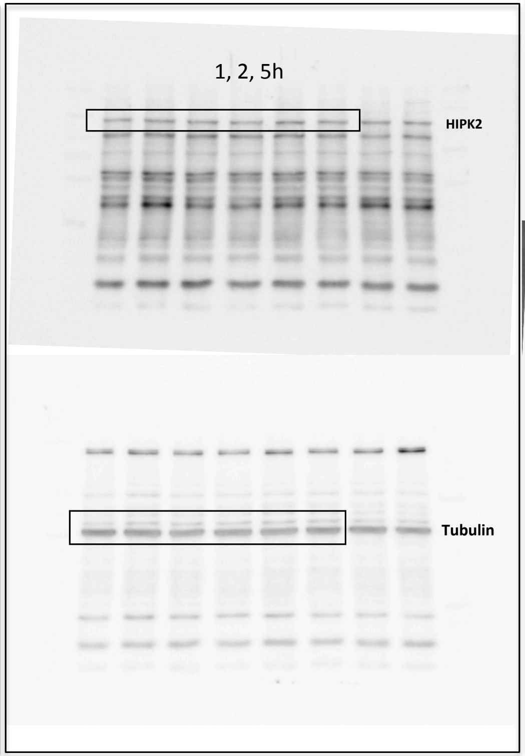

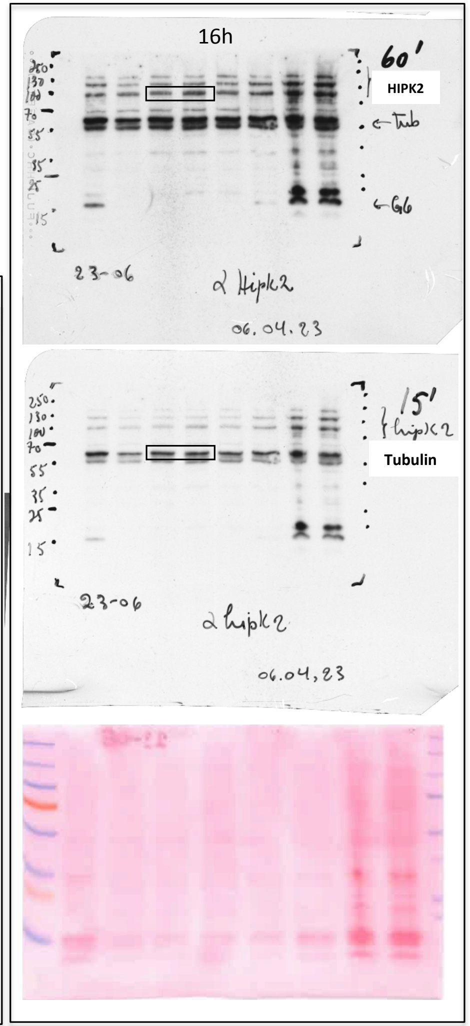

In figure 1G, FNIP1

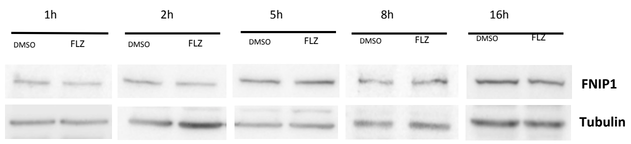

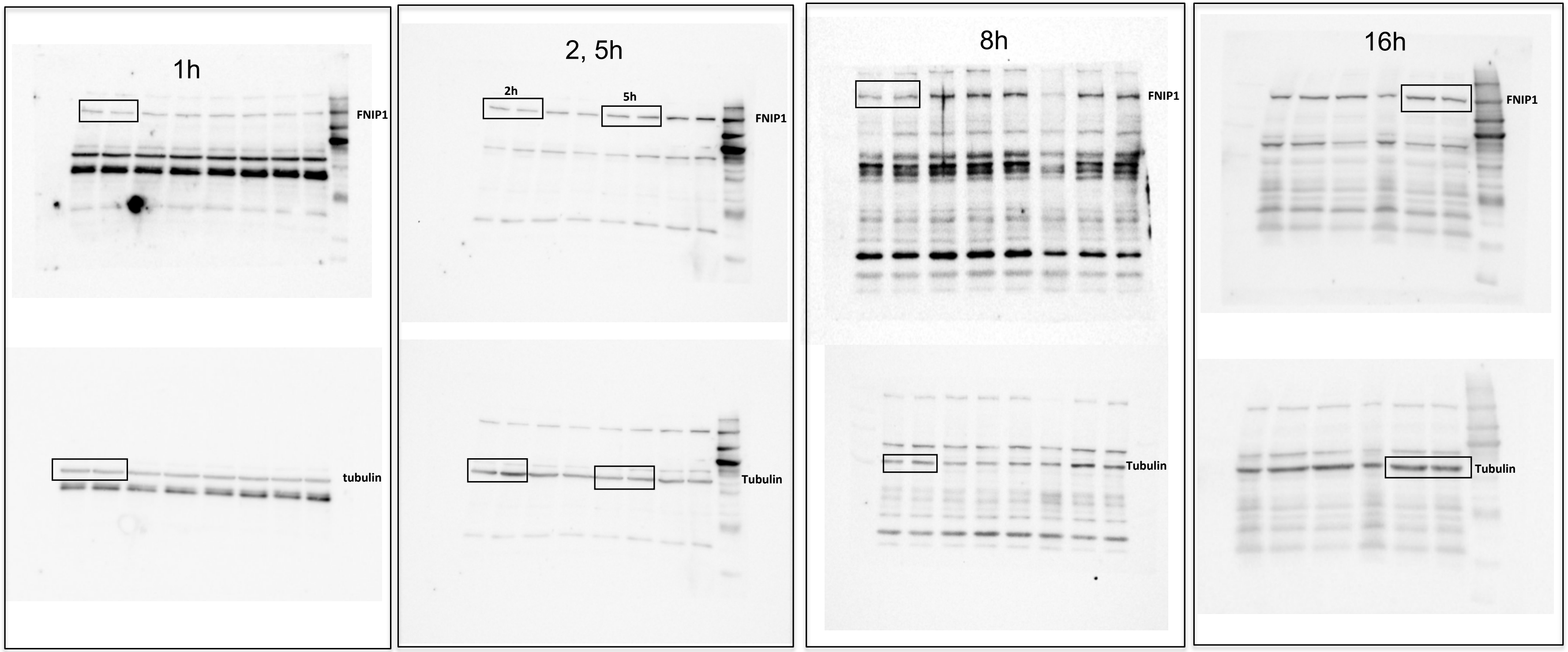

in figure 1G, FUS

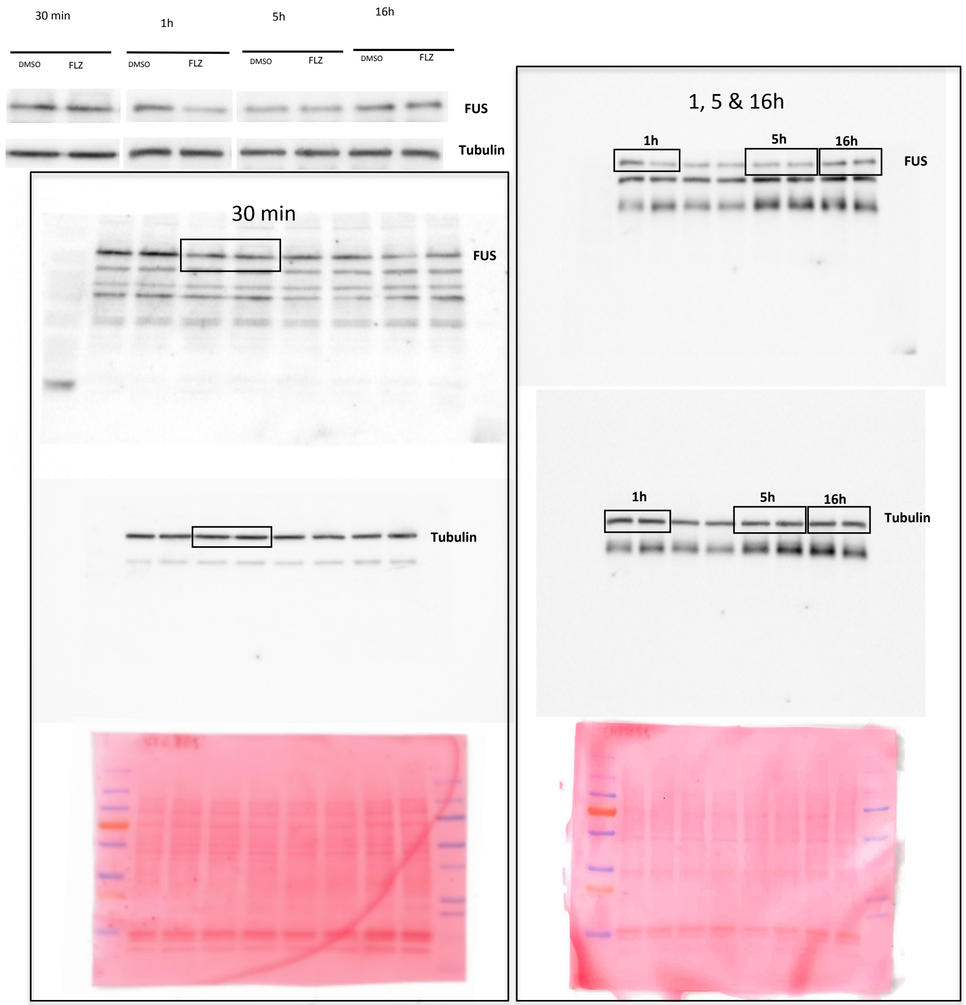

In figure 1G,TDP43

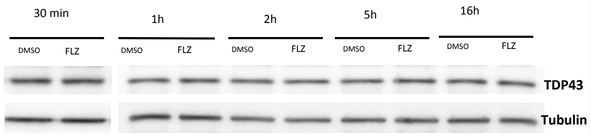

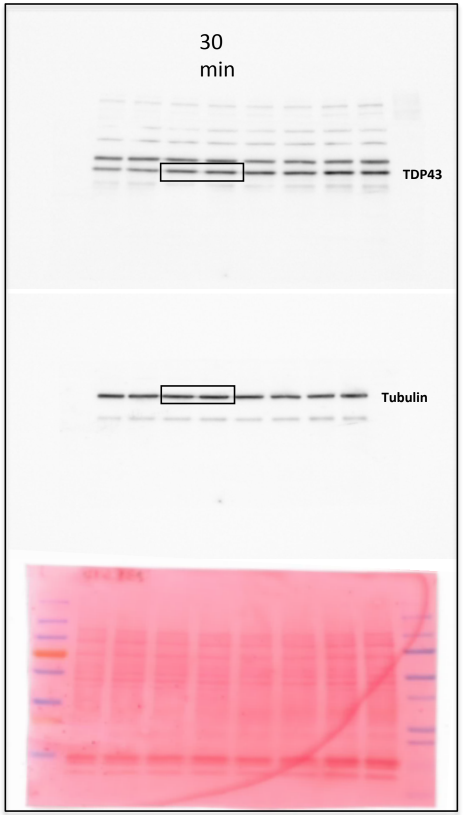

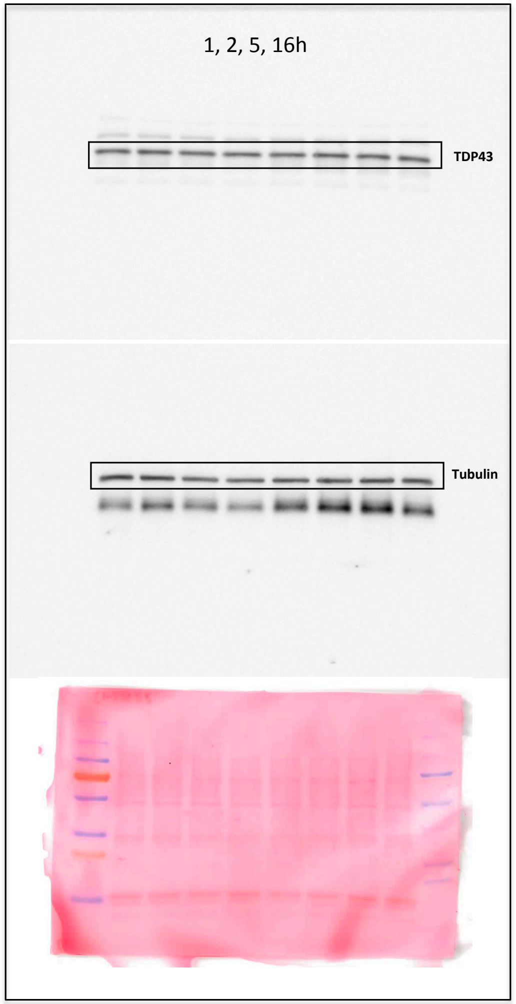

In figure 2A, first panel

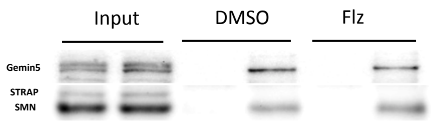

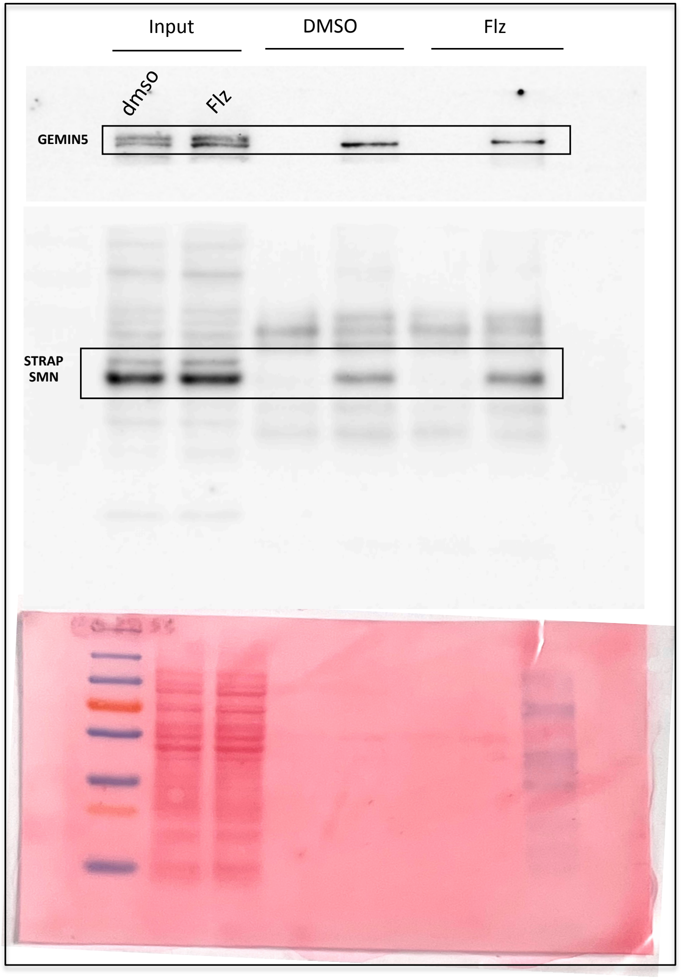

In figure 2A, second panel

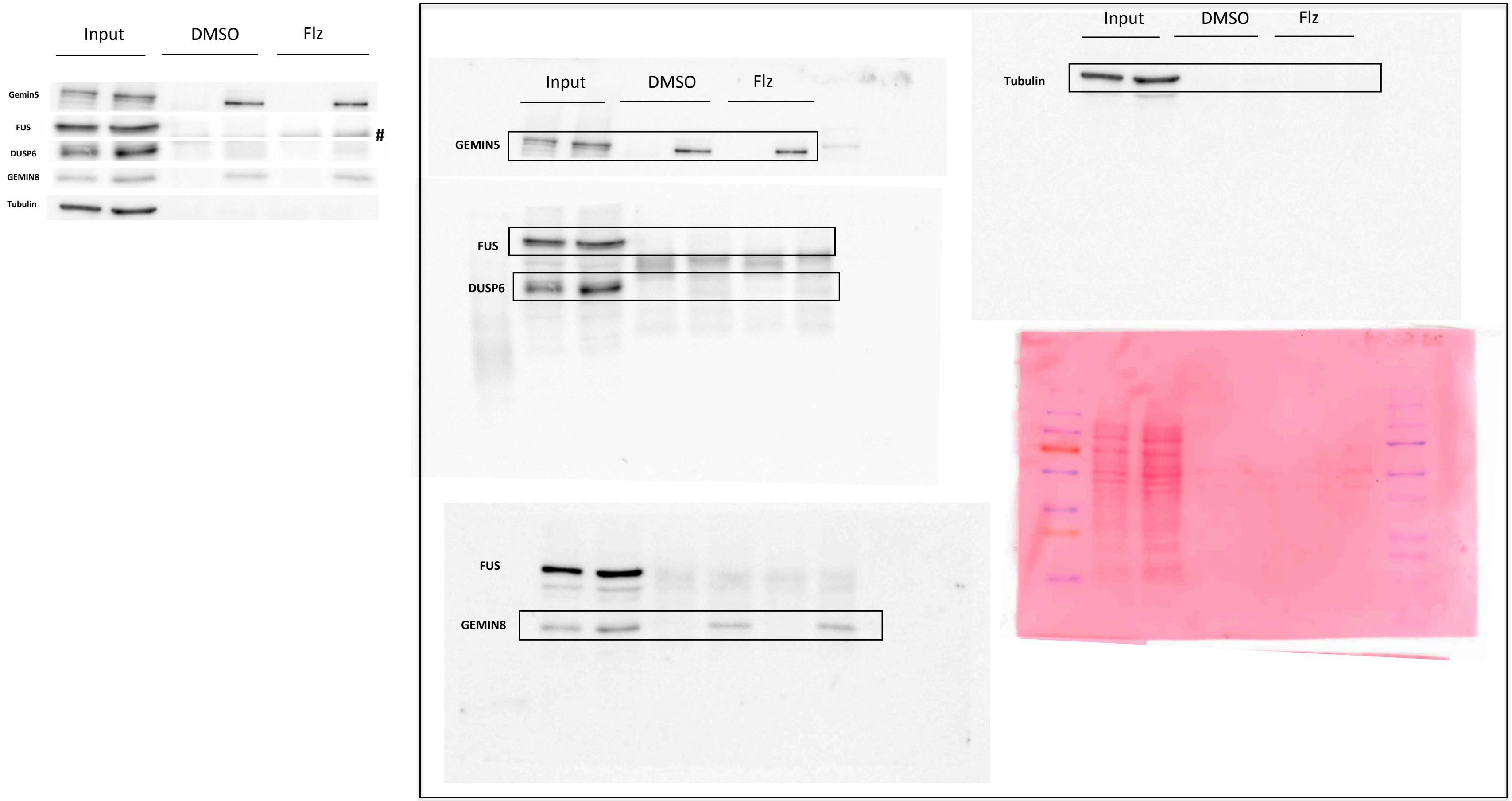

In figure 2A, third panel

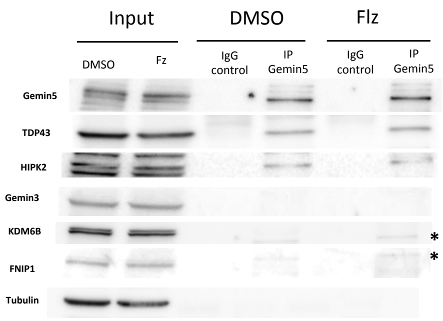

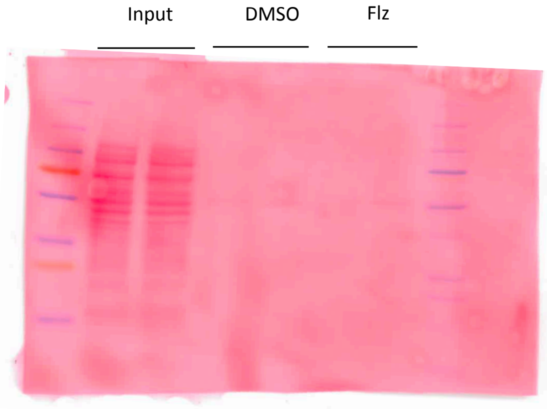

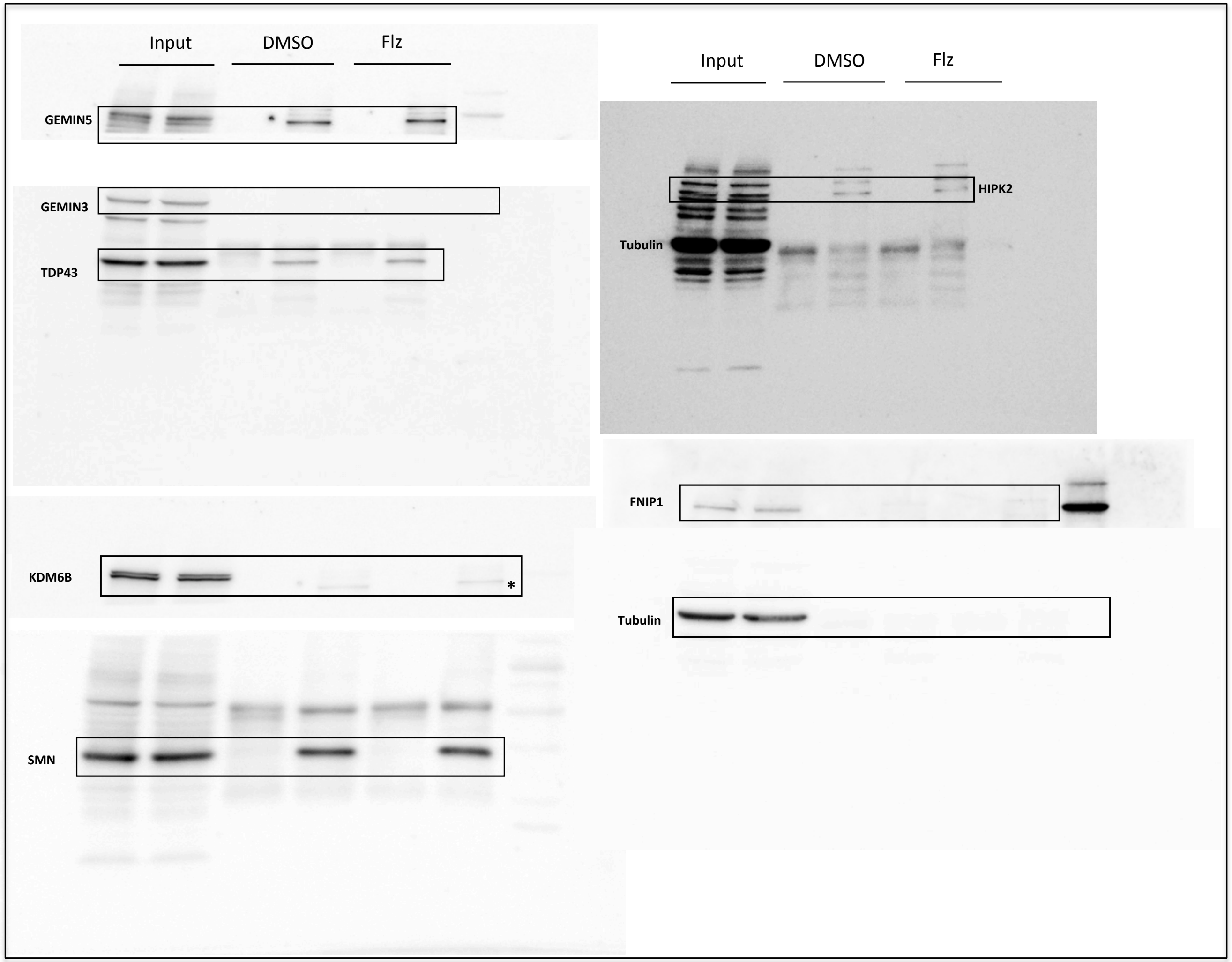

In figure 3E

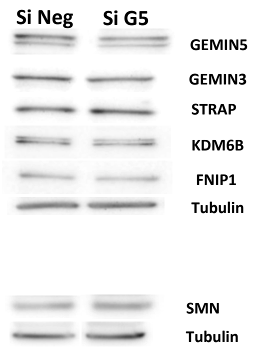

Si Neg
Si G5
GEMIN5
GEMIN3
STRAP
KDM6B
FNIP1
Tubulin
SMN
Tubulin

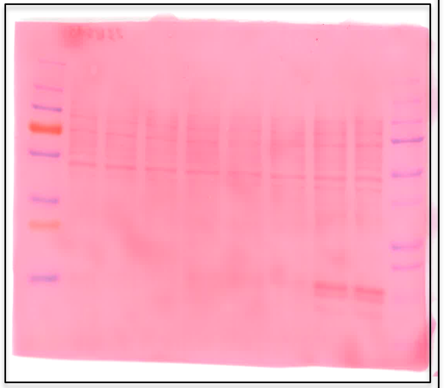

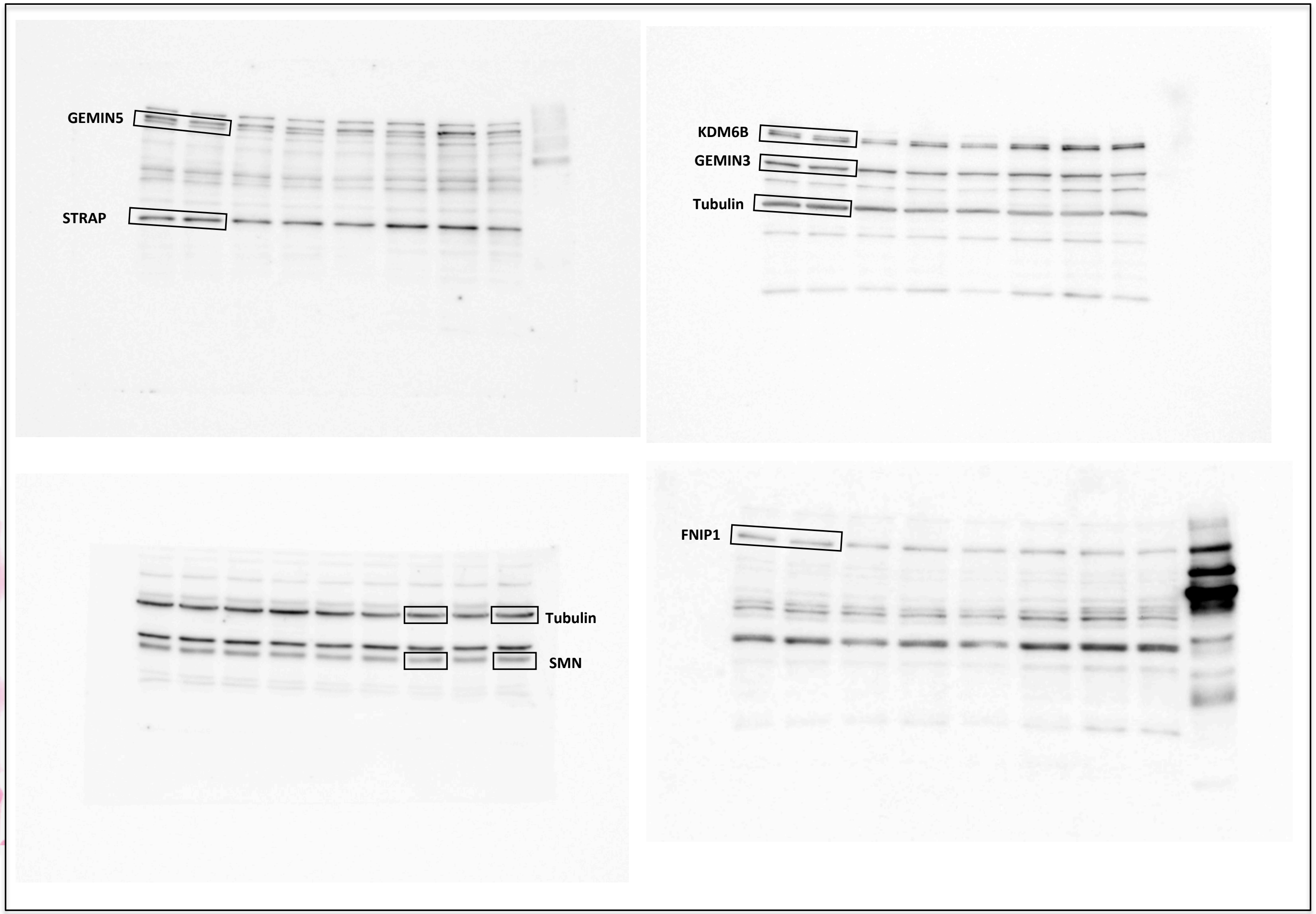

GEMIN5
STRAP
KDM6B
GEMIN3
Tubulin
Tubulin
SMN
FNIP1

in figure 3G

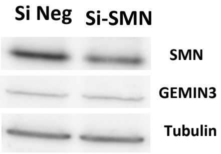

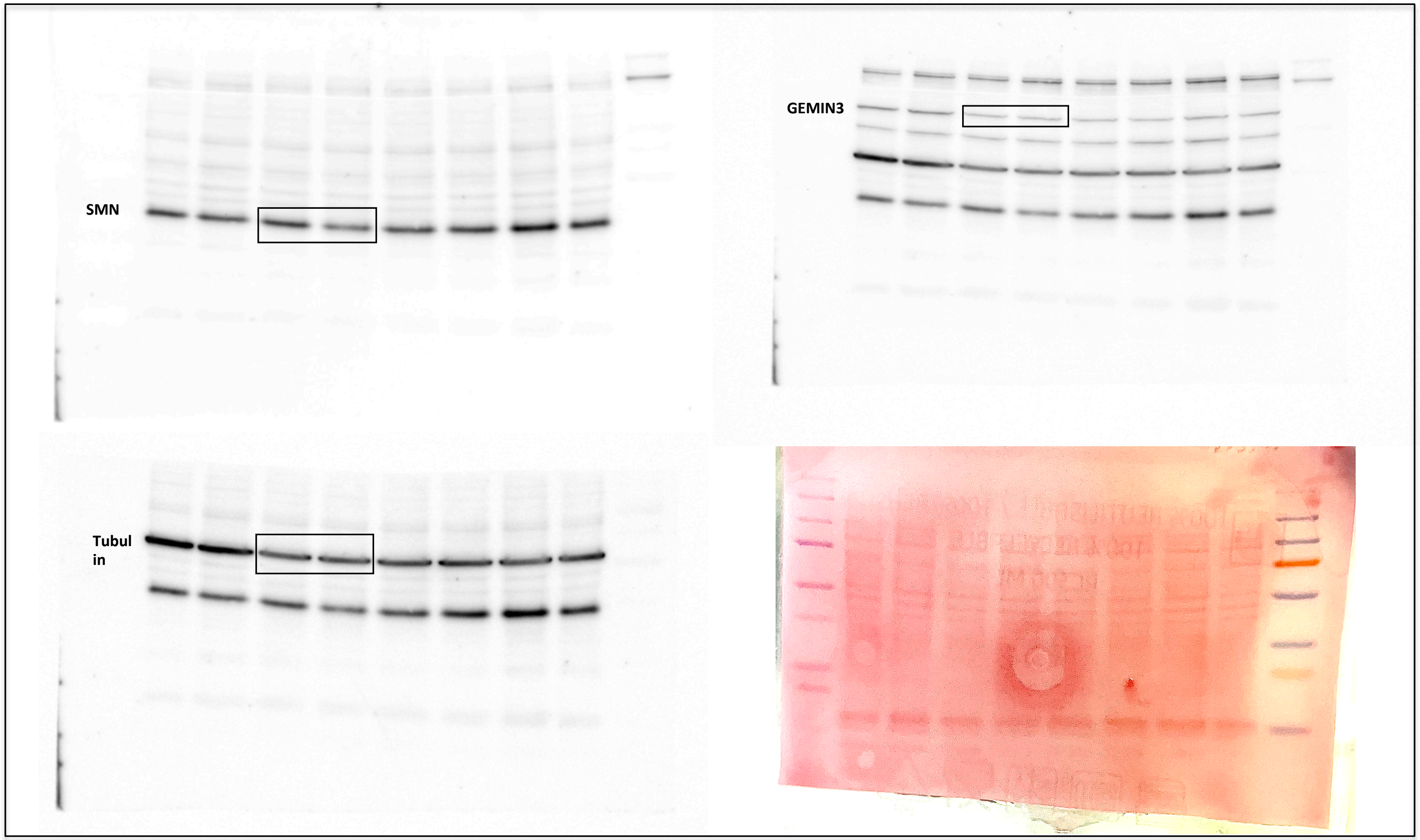

in figure 3G

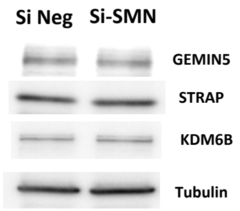
Si Neg
Si-SMN
GEMIN5
STRAP
KDM6B
Tubulin

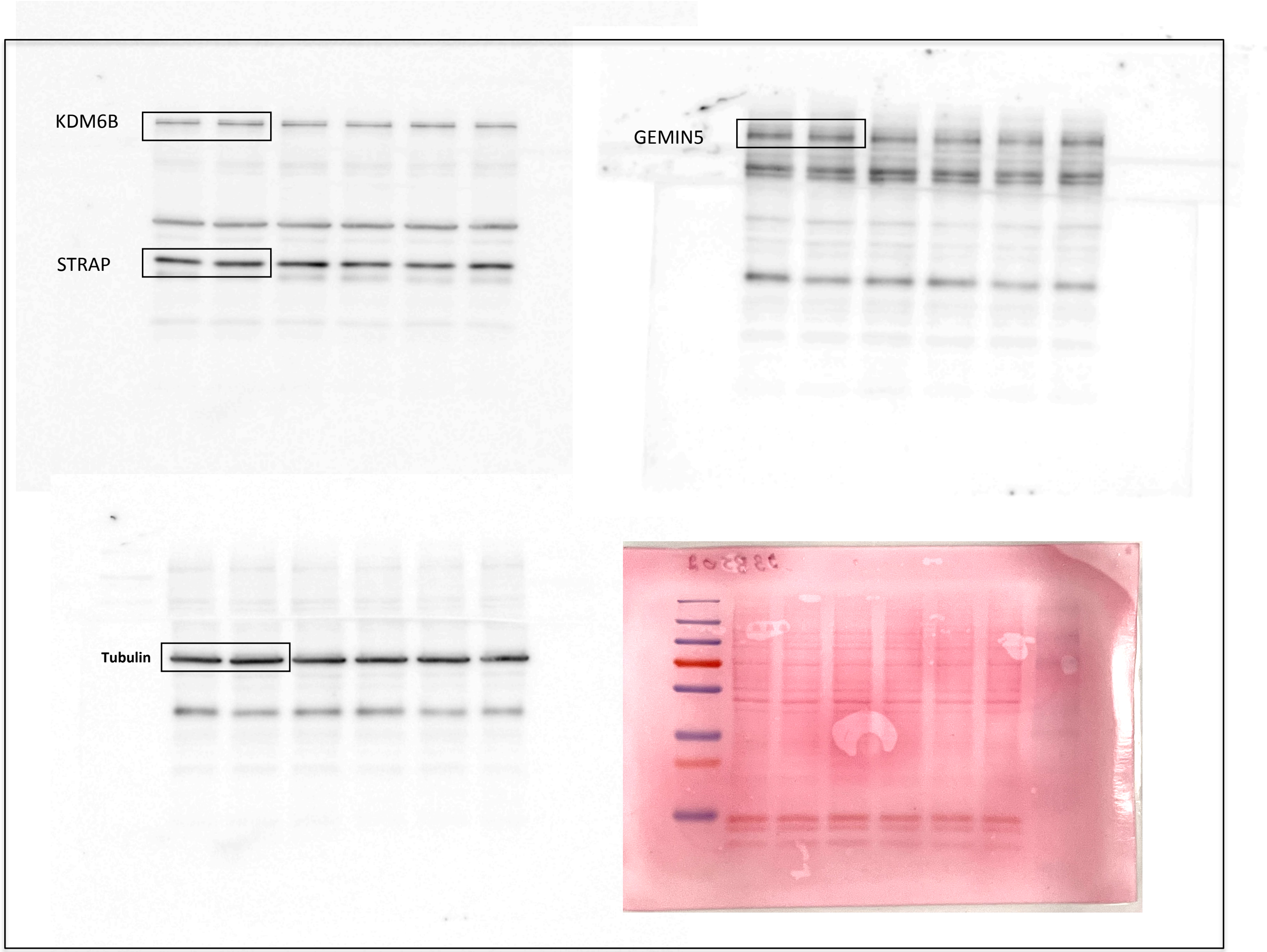
KDM6B
STRAP
GEMIN5
Tubulin

In figure 4D, HB9

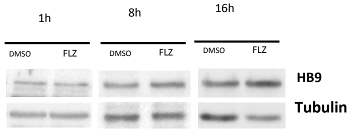

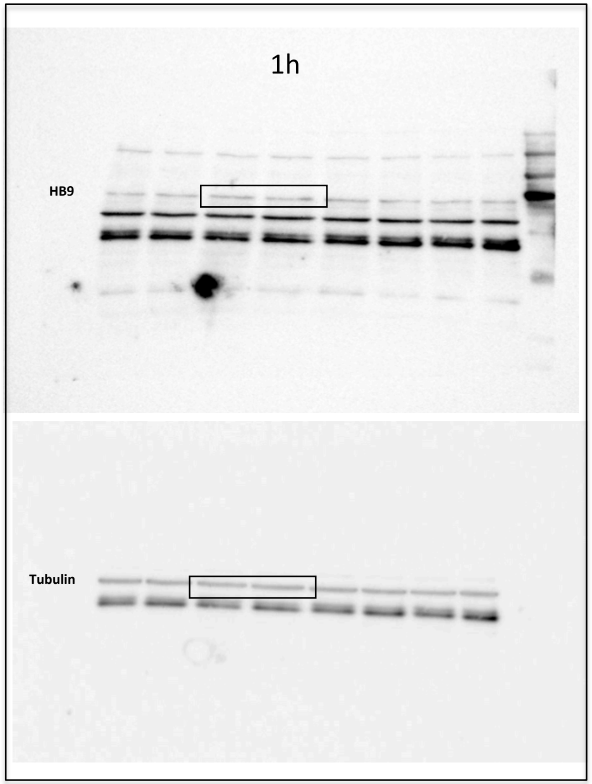

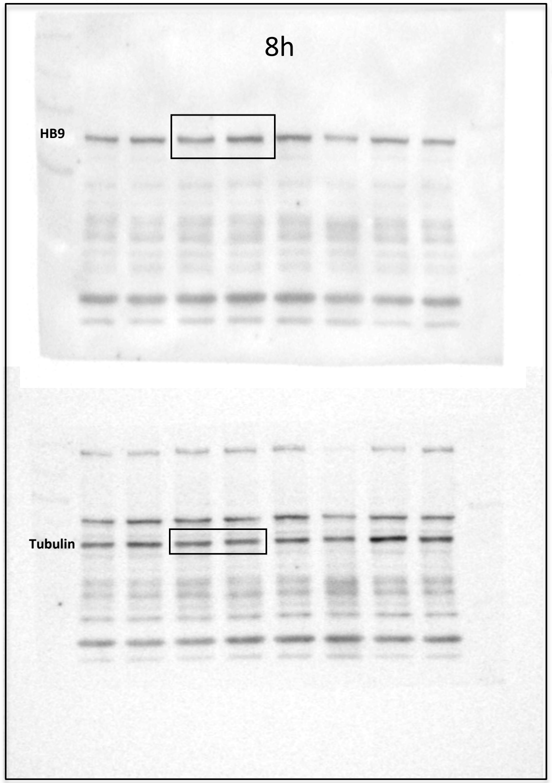

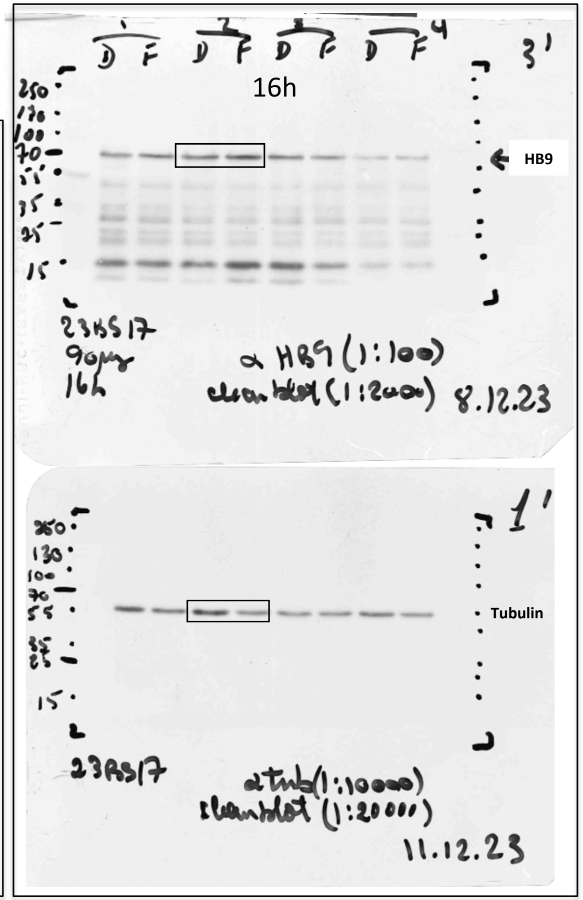

In supplemental figure S3

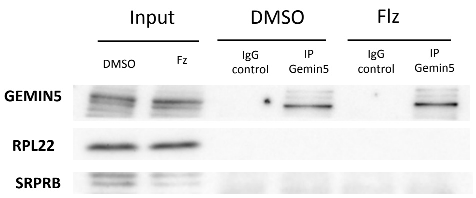

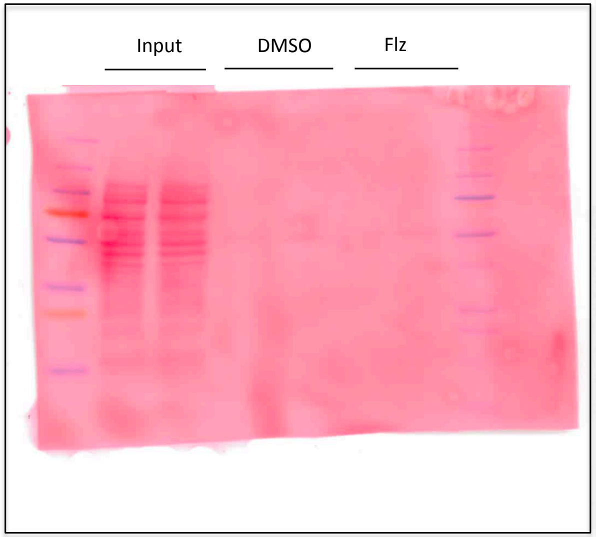

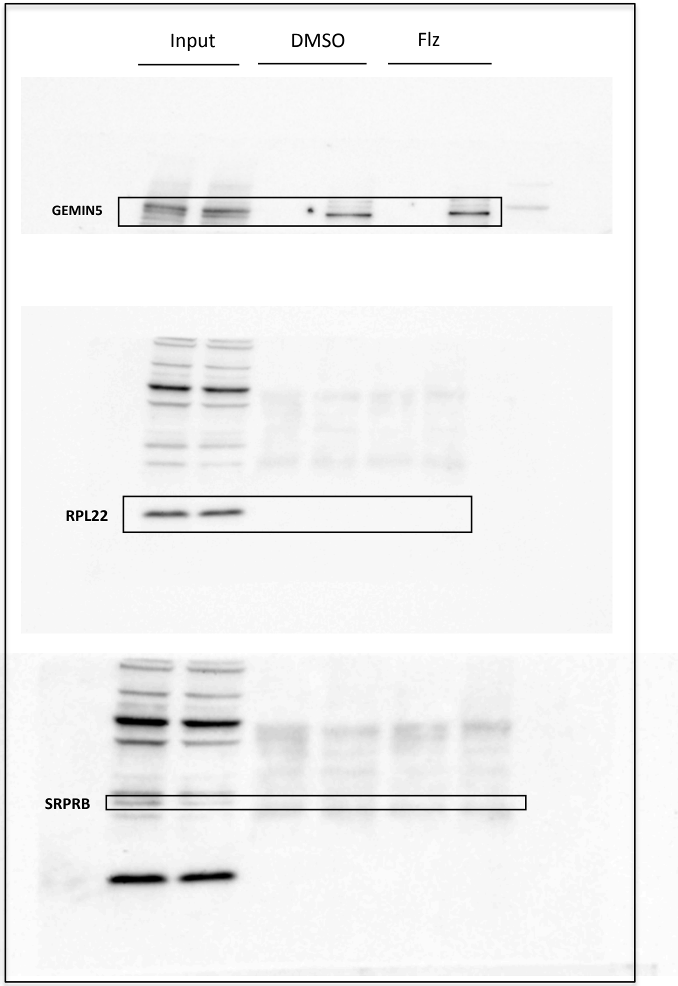

Supplement: Supplementary file 1 [file ijms-25-10039-s001.zip › ijms-3205342-supplementary.pdf]
